# Adverse Events of Mood Monitoring and Ambulatory Assessment in Depression and Bipolar Disorder: Systematic Review and Meta-Analysis

**DOI:** 10.2196/79500

**Published:** 2025-10-23

**Authors:** Laurence Astill Wright, Jonathan Monk-Cunliffe, Boliang Guo, Richard Morriss

**Affiliations:** 1 Institute of Mental Health, University of Nottingham, Innovation Park, Triumph Road, Nottingham, NG7 2TU, United Kingdom, 44 0115 823 1294; 2 Centre for Academic Mental Health, Population Health Sciences, University of Bristol, Bristol, United Kingdom; 3 NIHR ARC East Midlands, University of Nottingham, Nottingham, United Kingdom; 4 Nottingham NIHR Biomedical Research Centre, University of Nottingham, Nottingham, United Kingdom; 5 NIHR MindTech Medical Technology Collaborative, University of Nottingham, Nottingham, United Kingdom

**Keywords:** depression, bipolar disorder, digital mental health, ecological momentary assessment, EMA, mood monitoring, ambulatory assessment, mood tracking, adverse events, adverse effects, Preferred Reporting Items for Systematic Reviews and Meta-Analyses, PRISMA

## Abstract

**Background:**

Mood monitoring and ambulatory assessment offer improvements in measuring mood and behavior for mental health research and clinical practice. However, concerns about adverse effects and usability may hinder their implementation.

**Objective:**

This systematic review and meta-analysis assessed the prevalence of adverse events, barriers and facilitators to use, and suggestions for improvement in quantitative mood monitoring studies involving people with depression and bipolar disorder.

**Methods:**

We conducted a systematic review and meta-analysis of 77 quantitative studies that used mood monitoring or ambulatory assessment in depression and bipolar disorder, assessing adverse events, barriers and facilitators to use, and suggestions for improvement. Adverse events data were pooled to identify prevalence.

**Results:**

Of the 77 studies, 15 (19%) reported adverse events, and 20 (26%) reported usability issues. Pooled prevalence of adverse events was 0.04 (95% CI 0.03-0.06; *P*<.001). Specific adverse effects included increased burden or stress (0.04, 95% CI 0.02-0.07; *P*<.001), mood worsening (0.02, 95% CI 0.01-0.02; *P*=.001), self-harm (0.05, 95% CI-0.02 to 0.10; *P*=.007), and hospitalization (0.06, 95% CI 0.04-0.09; *P*=.26). The top facilitators were perceived helpfulness and ease of use, the top barriers included technical challenges and the time-consuming nature of the interventions, and the top suggested improvement was personalization.

**Conclusions:**

A small number of mood monitoring or ambulatory assessment users experienced negative psychological effects; however, we were unable to infer causality. Due to the severe underreporting of adverse events as well as heterogeneity and publication bias in the included studies, there was limited certainty in the prevalence, duration, and severity of these adverse events. More systematic monitoring of adverse events is needed to optimize safety and usability. Many mood monitoring protocols may require additional development to decrease adverse events and improve acceptability.

## Introduction

This review analyzes adverse effects associated with mood monitoring, mood tracking, and ambulatory assessment in people with unipolar depression and bipolar disorder (BD). Ambulatory assessment encompasses a diverse set of methods that use mobile technology to collect repeated measurements from participants, often in real time and in natural settings [1]. It includes mood monitoring, remote measurement technologies (eg, wearables that passively collect data), and ecological momentary assessment (EMA; a more intensive form of data collection involving multiple reports per day [2]). Some studies of mood monitoring also fall under EMA, remote measurement, or ambulatory assessment, and there is definitional overlap among these approaches. Mood monitoring can be used as an intervention (in both randomized controlled trials [RCTs] and nonrandomized studies) or as a method of outcome assessment (also in both RCTs and nonrandomized studies).

Technological advances (eg, wearables and smartphone sensors) have improved the potential of mood monitoring via novel methods of active (manual input) and passive (automatic) data collection [3]. These measures have many promising potential use cases, but smartphone-based ambulatory assessment tools may present unique risks, particularly in populations considered vulnerable [4]. While ambulatory assessment, mood monitoring, and EMA are performed in diverse populations for a wide variety of reasons (eg, dietary studies in healthy volunteers [5]), we chose to focus on people with unipolar depression and BD because evidence suggests that the risks in this population are unique [6,7]. Ambulatory assessment in psychiatric disorder also presents unique opportunities as well as challenges, given the difficulty of measuring rapidly shifting psychopathology with conventional measurement scales [8].

The risks of mood monitoring can be higher in self-guided apps, which have fewer built-in safety mechanisms to monitor user safety in real time [9]. Research on psychological treatments has historically focused on benefits rather than any negative psychological experiences [10]. Awareness of adverse events from internet- or app-based interventions is growing, and key academics have proposed consensus statements to guide future research on adverse events in this context [9,11,12]. This includes how to report, assess, and define adverse effects. The occurrence of adverse events may or may not be directly related to the treatment being used, and this review cannot assess causality [9,10]. Adverse effects of psychological interventions can range from mild experiences (eg, privacy concerns and frustration) to severe outcomes (eg, hospitalization, suicidality, and self-harm) [10].

Previous research has raised concerns that self-monitoring protocols could contribute to the maintenance of depression in some participants due to a variety of mechanisms [6,13,14]. The protocols could worsen negative processing bias, as participants confront their distressing experiences on a daily basis. It could be a painful reminder of their mental health problems, for example, or cause them distress if they realize that their mood is worsening and feel powerless to change it. The process could also be a time-consuming burden to them that diverts energy from more therapeutic activities. This could partially explain the occasionally occasionally high attrition rates and poor adherence observed in some interventions [15]. There has been little research examining adverse events from ambulatory assessment use or self-monitoring activities. Fundamental questions remain about the negative effects of mood monitoring interventions and how they might be improved to maximize their usefulness for people with depression or BD. Mixed methods research offers good potential to elucidate some of these factors [16,17].

It is important to consider the risks to maximize the potential benefits of these advanced technologies. The promising potential applications of these electronic tools are summarized briefly here. They may improve on existing research methods by decreasing participant burden and allowing for a higher quality and granularity of mood assessment at decreased economic cost. They may also improve the recall bias associated with some measures of psychopathology [18]. They may help in the treatment of depressive illness by detecting early behavioral change and allowing earlier intervention, potentially allowing individuals with depression to self-manage their condition. They may also help define clinical goals collaboratively with clinicians or be used as research tools to identify digital depressive phenotypes to potentially improve treatment personalization in mental health care. While compliance and acceptability have been studied extensively [7,15,19], there is limited understanding of the adverse effects of these ambulatory assessment approaches in clinical mental health care or research [20].

This is the first systematic review, to our knowledge, that assesses and quantifies adverse events in ambulatory assessment and mood monitoring studies. Specifically, we aimed to quantify the prevalence of adverse events and explore barriers and facilitators to use as well as any suggested improvements to identify common themes that may aid or hinder engagement with mood monitoring protocols. We chose to examine adverse events in studies where mood monitoring was used as an intervention (in both RCTs and nonrandomized studies) or as a method of outcome assessment (also in both RCTs and nonrandomized studies) because both these use cases are important to people with BD and depression, and both use cases have reported qualitative data on adverse events [6]. As we reviewed evidence of varying types, we are unable to make any definitive comments on the causality of the adverse events.

## Methods

We used a methodology based on the Cochrane Handbook for Systematic Reviews of Interventions and completed a PRISMA (Preferred Reporting Items for Systematic Reviews and Meta-Analyses) checklist (Checklist 1). The study was preregistered with PROSPERO (CRD42023396473) [21].

### Inclusion Criteria

We included studies if they met the following criteria: they involved self-monitoring, EMA, or repeated symptom assessment in people with BD or depression over a minimum period of 3 months, with symptom ratings conducted at least weekly. Each study was required to use a validated measure of mood or to validate the chosen measure against an established mood measure. Studies published in any language were eligible and could be either digital or nondigital, although we anticipated that the majority of studies would use digital technologies. We included RCTs or nonrandomized studies with 20 or more participants with BD or depression [22]. We chose a cutoff of 20 participants to include mood monitoring protocols that were closer to clinical implementation (eg, the National Institute for Health and Care Excellence frequently excludes studies of fewer than 20 participants in its reviews and guideline documents). Studies with fewer than 20 participants were also considered unlikely to have sufficient data on adverse events. We searched gray literature (eg, conference abstracts, dissertations, policy literature, and reports identified through ProQuest and Google Scholar [for details, refer to the next subsection]) for unpublished studies that were eligible for inclusion.

### Search Strategy and Selection Criteria

The complete search strategy is presented in Multimedia Appendix 1. We searched Ovid MEDLINE, Embase, PsycInfo, Scopus, IEEE Xplore, ProQuest SciTech Premium Collection, ProQuest Dissertations & Theses Global, and Google Scholar using the defined search terms. The initial search was conducted on March 3, 2023, and updated on October 28, 2024 [23]. All abstracts were appraised by two screeners, and any disagreements were discussed until consensus was reached, with adjudication by a third independent screener if required. The full text of potentially relevant papers was retrieved, and if unavailable, the corresponding author was contacted. To determine whether potentially relevant studies met the inclusion criteria, the full text was reviewed separately by two authors, again resolving discrepancies through discussion or, if necessary, consultation with a third author. Reference lists of included studies and relevant systematic reviews [13,14,24-31] were also screened to identify additional eligible studies. Key authors were contacted to inquire about any ongoing or unpublished studies that might meet the inclusion criteria.

### Data Extraction

Two independent reviewers extracted data from studies meeting the inclusion criteria using identical data extraction forms. Any irregularities in the data extraction were discussed, and discrepancies were resolved through discussion. No deviation from the protocol registered with PROSPERO occurred. We defined an adverse event as any untoward or unintended medical occurrence in a participant, regardless of whether it was related to the intervention or investigation [32]. All adverse events were included in the results and assessed for relatedness to the mood monitoring protocol by two psychiatrists. Those deemed possibly related were then meta-analyzed. Because of confounding factors in the expected psychiatric deterioration in individuals with relapsing-remitting conditions, we were not able to make definitive inferences about adverse event causality.

### Assessment of Study Bias

The Cochrane risk-of-bias tool (RoB 2) was used for RCTs [33], and the Cochrane risk of bias in nonrandomized studies of interventions tool (ROBINS-I) [34] was used for nonrandomized studies. Risk of bias was assessed by two independent reviewers, and any disagreements were resolved via discussion.

### Synthesis of Results

For quantitative data, studies were grouped, where possible, according to the variable assessed (eg, adverse events such as self-harm or subjective worsening of mood), and data were pooled in a meta-analysis. We conducted a separate analysis for specific adverse events because we hypothesized that their prevalence would vary. The results of each primary study were pooled by means of an inverse variance–weighted approach with random or fixed effects models, informed by examination of between-study heterogeneity. Stata *metan* code was used to perform the analysis for proportion data. Comparisons with only 2 studies were excluded from any separate analysis.

For qualitative data, papers were read and reread by two independent reviewers. We assessed qualitative reporting of adverse events in the quantitative papers because we hypothesized that quantitative reporting might be limited [12]. Second-order constructs were extracted and managed using Microsoft Excel. Any disagreements were discussed until consensus was reached. Constructs were reviewed to assess how the themes juxtaposed and compared across studies. Reviewers independently reviewed the second-order constructs [35] and compiled third-order constructs [35,36] that summarized and encapsulated the various themes across the studies using NVivo 12 (Lumivero) [37]. These constructs were then refined through discussion between researchers until a shared understanding was reached.

The guidelines for meta-ethnography outlined by Noblit and Hare [38] were used to conduct the analysis. Noblit and Hare [38] proposed three ways to synthesize data: (1) reciprocal translation (where the findings of one study are understood in terms of findings expressed in other studies in the synthesis [39]) if the data are directly comparable; (2) refutational translation (which explains and explores inconsistencies, exceptions, and incongruities in the data between studies [40]) if the data are in opposition; and (3) an integrating approach that makes sense of the parts—a “line of argument” synthesis that uses both similarities and differences across the studies. Our assessment of the included studies showed consistent themes in terms of the adverse events reported in mood monitoring and ambulatory assessment protocols. Therefore, we used the “line of argument” approach to make sense of apparent contradictions in the data before integrating the emergent concepts into a framework of reported adverse events.

## Results

### Overview

**Figure 1.**
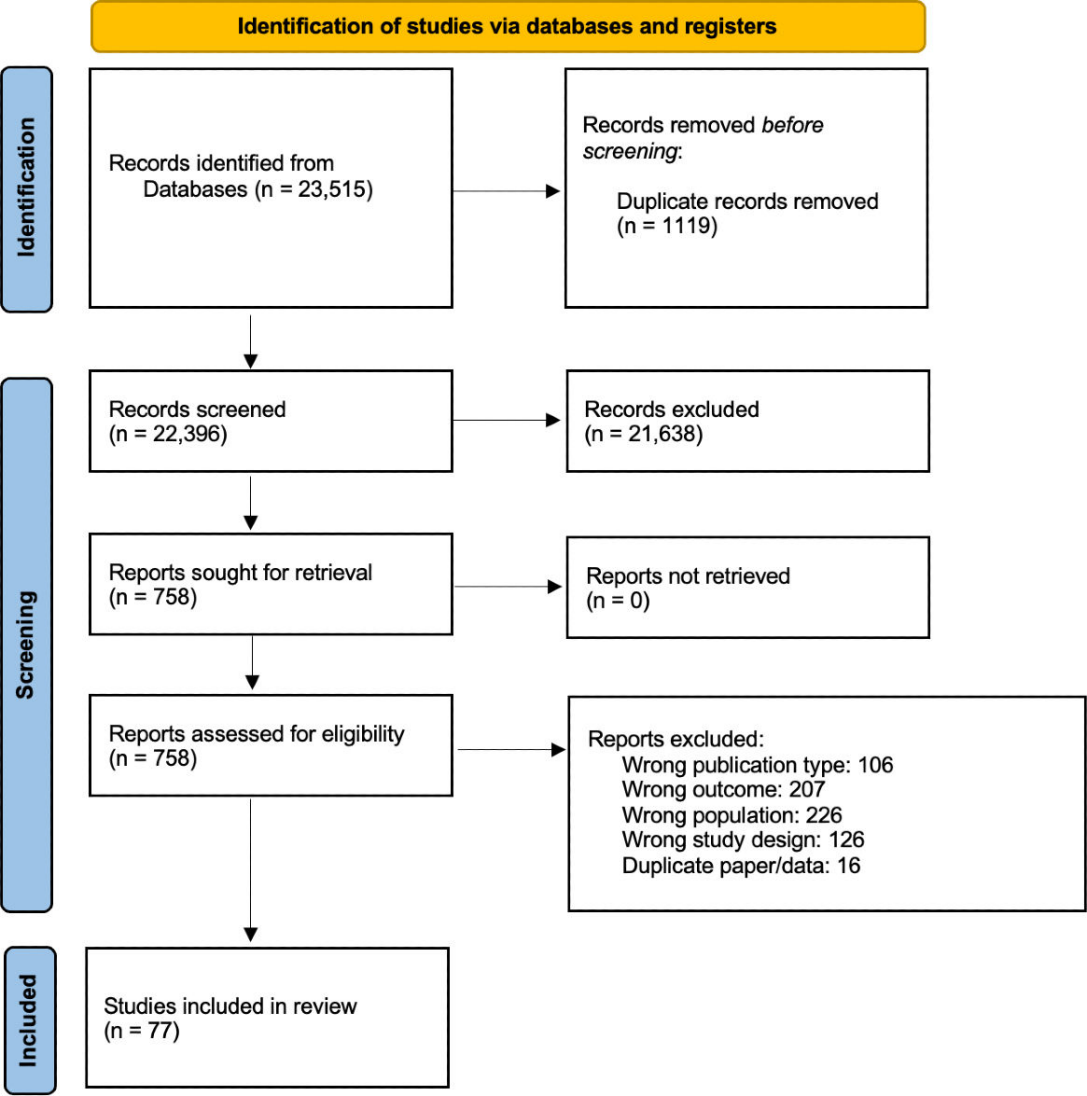
PRISMA (Preferred Reporting Items for Systematic Reviews and Meta-Analyses) flow diagram showing the number of studies identified, screened, assessed for eligibility, and included in the final analysis.

The search identified 23,515 papers, of which 1119 (4.76%) were duplicates and removed. There were no studies reported in languages other than English that met the inclusion criteria. After title and abstract screening, 21,638 (96.61%) of the 22,396 papers were excluded, resulting in a total of 758 (3.5%) papers being reviewed in full. Of these 758 papers, 77 (10.2%) met the eligibility criteria. Of these 77 papers, 33 (43%) reported adverse events, usability issues, or feasibility issues and were included in the analyses. The PRISMA flow diagram is presented in Figure 1.

The 77 included studies included 16,165 participants, and the 33 studies with usable data included 4949 participants. Tables S1 and S2 in Multimedia Appendix 1 present detailed characteristics of the studies and the mood monitoring protocols used, Table S3 in Multimedia Appendix 1 reports risk-of-bias assessments, and Table S4 in Multimedia Appendix 1 reports adverse event data. Tables1^3^ present facilitators, barriers, and suggested improvements, respectively, for mood monitoring protocols.

**Table 1.** Facilitators in the included studies (n=24).

Facilitators	Studies reporting, n (%)	Condition (bipolar disorder or depression)	Studies reporting
It is helpful	9 (38)	Bipolar disorder and depression	Hidalgo-Mazzei et al [41]; Hidalgo-Mazzei et al [42]; Garcia-Estela et al [43]; van den Heuvel et al [44]; de Angel et al [45]; Van der Watt et al [46] ; Osgood-Hynes et al [47]; Sharp et al [48]; Pahwa et al [49]
Ease of use	10 (42)	Bipolar disorder and depression	Hidalgo-Mazzei et al [41]; Hidalgo-Mazzei et al [42]; Garcia-Estela et al [43]; Bowden et al [50]; van den Heuvel et al [44]; Drake et al [51]; Janevic et al [52]; Matcham et al [53]; Faurholt-Jepsen et al [54]; Pahwa et al [49]
Aided medication adherence	2 (8)	Depression	Janevic et al [52]; Van der Watt et al [46]
Improved insight	2 (8)	Depression	Janevic et al [52]; Van der Watt et al [46]
Sharing with friends and family	1 (4)	Depression	Drake et al [51]
Sharing with clinician	1 (4)	Bipolar disorder	van den Heuvel et al [44]
Variety of questions	1 (4)	Depression	Bonilla-Escribano et al [55]
Smartphone was provided	1 (4)	Bipolar disorder	Faurholt-Jepsen et al [54]

**Table 2.** Barriers in the included studies (n=24).

Barriers	Studies reporting, n (%)	Condition (bipolar disorder or depression)	Studies reporting
Technical issues	9 (38)	Bipolar disorder and depression	Hidalgo-Mazzei et al [41]; Hidalgo-Mazzei et al [42]; van den Heuvel et al [44]; Janevic et al [52]; Matcham et al [53]; Meyerhoff et al [56]; Faurholt-Jepsen et al [57]; Lauder et al [58]; Goulding et al [59]
Time consuming	9 (38)	Bipolar disorder and depression	Hidalgo-Mazzei et al [42]; Garcia-Estela et al [43]; van den Heuvel et al [44]; de Angel et al [45]; Drake et al [51]; Faurholt-Jepsen et al [54]; Lauder et al [58]; Goulding et al [59]; Bowden et al [50]
Repetitive	4 (17)	Bipolar disorder and depression	Hidalgo-Mazzei et al [42]; Garcia-Estela et al [43]; de Angel et al [45]; Janevic et al [52]
Intervention not useful	6 (25)	Bipolar disorder and depression	Hidalgo-Mazzei et al [41]; Hidalgo-Mazzei et al [42]; Garcia-Estela et al [43]; Drake et al [51]; Van der Watt et al [46]; Matcham et al [53]
Unwelcome reminder of mental illness	2 (8)	Bipolar disorder	Hidalgo-Mazzei et al [42]; Garcia-Estela et al [43]
Difficult to use	3 (12)	Bipolar disorder and depression	Garcia-Estela et al [43]; Bowden et al [50]; Van den Heuvel et al [44]
Worsening of mood or anxiety	7 (29)	Bipolar disorder and depression	Drake et al [51]; Meyerhoff et al [56]; Bilderbeck et al [60]; Lauder et al [58]; White et al [61]; Tønning et al [62]; de Angel et al [45]
Burden of mood monitoring	5 (21)	Bipolar disorder and depression	van den Heuvel et al [44]; Matcham et al [53]; Helmich et al [63]; Janevic et al [52]; Smit et al [64]
Concern about stigma	1 (4)	Bipolar disorder	Garcia-Estela et al [43]
Privacy concerns	5 (21)	Bipolar disorder and depression	Hidalgo-Mazzei et al [42]; Garcia-Estela et al [43]; Helmich et al [63]; Matcham et al [53]; Meyerhoff et al [56]
Sharing with friends and family	1 (4)	Depression	Drake et al [51]
Wearables inconvenient	2 (8)	Depression	de Angel et al [45]; White et al [61]

**Table 3.** Suggested improvements and features in the included studies (n=24).

Suggested improvements and features	Studies reporting, n (%)	Condition (bipolar disorder or depression)	Studies reporting
Personalization	3 (12)	Bipolar disorder and depression	Hidalgo-Mazzei et al [41]; Hidalgo-Mazzei et al [42]; Garcia-Estela et al [43]
More human contact	1 (4)	Depression	Janevic et al [52]
Viewing mood trends over time	1 (4)	Bipolar disorder	Hidalgo-Mazzei et al [41]
Medication reminders	1 (4)	Bipolar disorder	Hidalgo-Mazzei et al [41]
Sharing with friends and family	1 (4)	Bipolar disorder	Hidalgo-Mazzei et al [41]
Gamification	1 (4)	Bipolar disorder	Hidalgo-Mazzei et al [41]
Psychoeducation	2 (8)	Bipolar disorder	Hidalgo-Mazzei et al [42]; Garcia-Estela et al [43]
Notifications to complete assessments	1 (4)	Bipolar disorder	Bowden et al [50]
Additional monitoring (other than mood)	1 (4)	Bipolar disorder	van den Heuvel et al [44]
Choosing when to complete assessment	2 (8)	Bipolar disorder and depression	Bowden et al [50]; Drake et al [51]
Easy-to-understand data	1 (4)	Bipolar disorder	Bowden et al [50]
Sharing with clinicians	1 (4)	Bipolar disorder	van den Heuvel et al [44]

The 77 included studies used 54 different mood monitoring protocols, and these are reported in Tables S1 and S2 in Multimedia Appendix 1. Follow-up periods varied from 3 months [65] to 3 years [66]. All studies included participants with depression and BD; however, 3 (4%) of the 77 studies also included a mixed sample (individuals with other diagnoses were included) [55,67,68] (Tables S1 and S2 in Multimedia Appendix 1). All studies used adult samples, apart from the study by Webb et al [69], which used a sample of adolescents.

Of the 77 studies, 15 (19%) reported adverse events, and 20 (26%) reported acceptability or usability issues (Table S1 in Multimedia Appendix 1). In some situations, usability issues or barriers to use and adverse events overlapped (eg, worsening of mood and burden of mood monitoring). Only 3 (4%) of the 77 studies used validated outcome assessments to measure adverse events [70-72]. The studies that reported adverse events (15/77, 19%) [44,51,53,55,57,59,60,62-64,71,73-76] did so via attrition numbers, which do not capture the number of people who experienced adverse events but remained in the study. Of the 77 studies, 13 (17%) [54,57,60,62,63,68,72,77-81] explicitly reported whether a deterioration in mood had occurred.

Only 2 (3%) of the 77 studies [61,82] explicitly reported an absence of adverse events. A total of 25 adverse events that varied from mild to severe were reported in the included studies (15/77, 19%). These were classified according to relatedness to the mood monitoring protocol (Table S4 in Multimedia Appendix 1), and those that were possibly related and included 3 or more studies were meta-analyzed. The studies by Smit et al [64] (which reported worsening of mood in the context of antidepressant discontinuation) and Helmich et al [63] (which reported negative psychological symptoms in the context of receiving psychological therapy) were excluded from the meta-analysis due to these confounding factors in the relatedness of the adverse events and the ambulatory assessment. Furthermore, studies reporting relapse were not meta-analyzed because the rates for relapse (40%‐60%) were considered too high to be related to the mood monitoring.

Of the 77 studies, 15 (19%) reported severe adverse effects, some of which were thought to be possibly related to the intervention (Table S4 in Multimedia Appendix 1). Of the 77 studies, 15 (19%) reported hospitalization [42-44,54,55,57,59,60,62,70,80,81,83-85]. Some of the studies measured hospitalization but did not report this as an adverse event; in many studies of BD, relapse and hospitalization are seen as an unfortunate part of the disease process. Of the 77 studies, 3 (4%) [44,55,60] reported hospitalization as an adverse event, and these were included in the meta-analysis because they were deemed to be possibly related to mood monitoring.

### Meta-Analysis

#### Total Prevalence of Adverse Events

**Figure 2.**
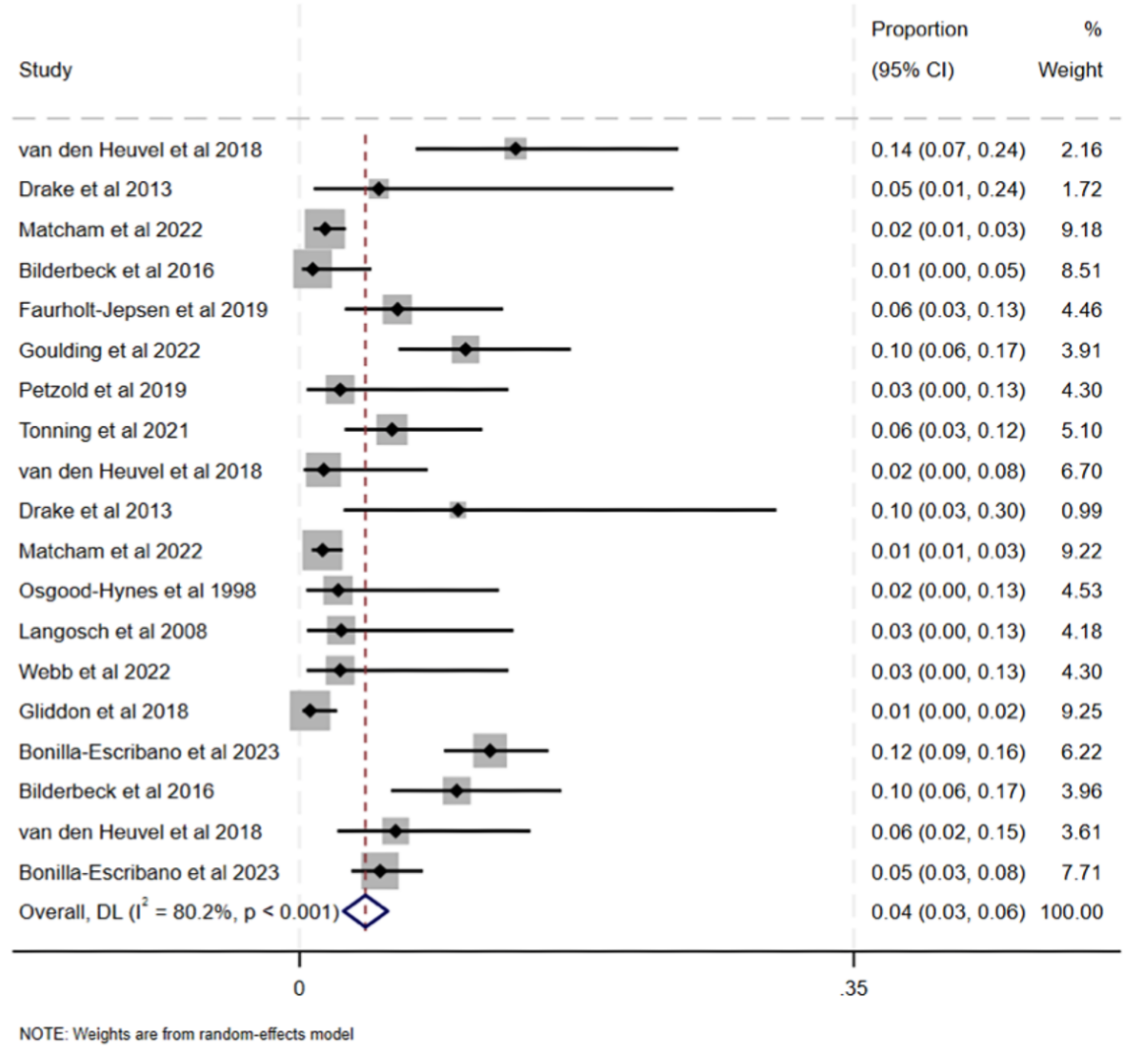
Forest plot of prevalence of adverse events with percentage weighting [44,47,51,53,55,59,60,62,70,71,74,76,80].

Pooled prevalence of adverse events was 0.042 (95% CI 0.028-0.056; *P*<.001; Figures2  3). Heterogeneity was high (Cochran Q_18_=91.00; *P*<.001; *I*^2^=80.2%), demonstrating large between-study variability. Because of the pronounced heterogeneity, we used a random effects model to pool the data. The funnel plot for the total prevalence of adverse events demonstrated visual asymmetry, suggesting possible publication bias and small study effects in the meta-analytic estimate (Figure S1 in Multimedia Appendix 1). The Egger test demonstrated that there were small study effects (Figure S2 in Multimedia Appendix 1), suggesting possible publication bias.

**Figure 3.**
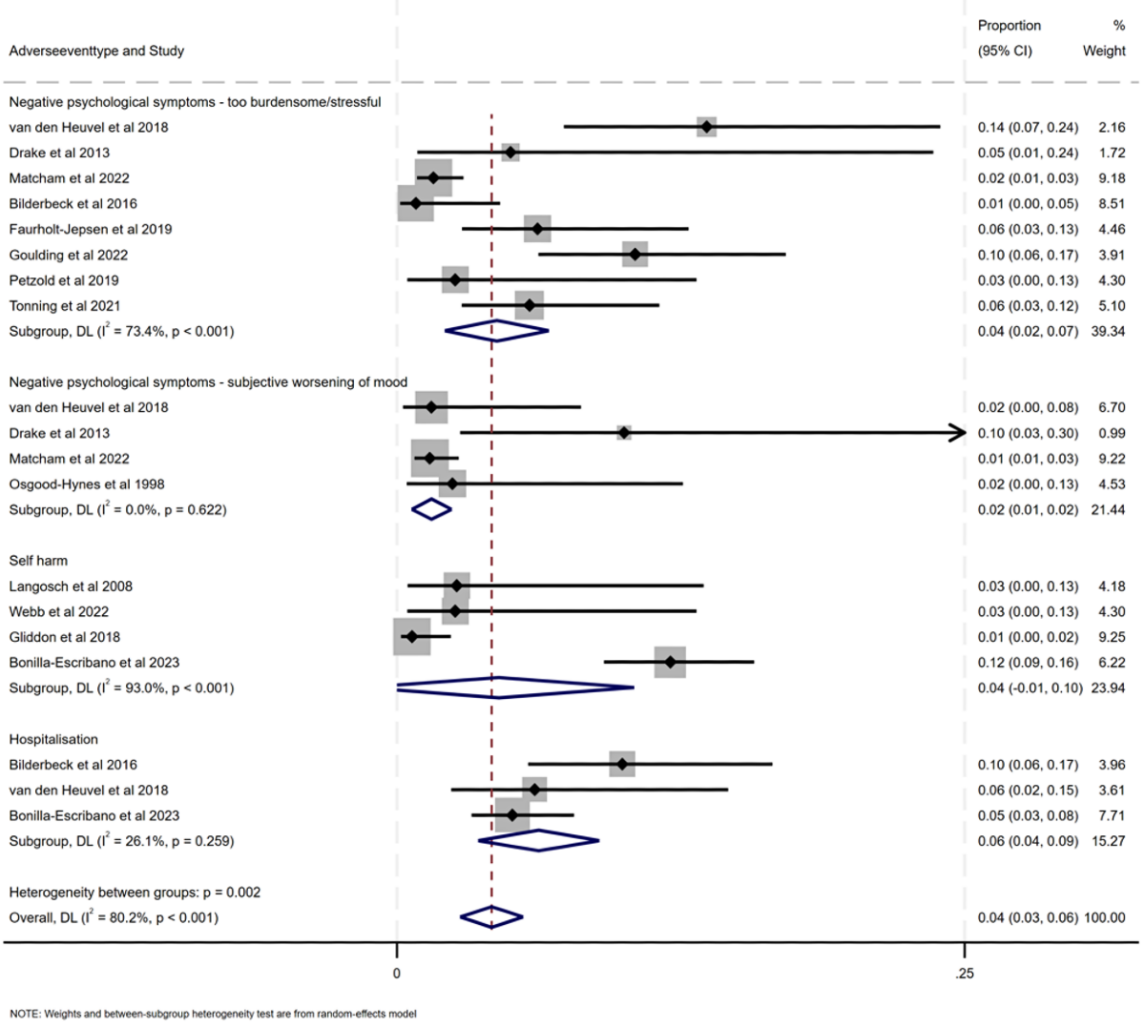
Forest plot of prevalence of adverse events stratified by type of adverse event (negative psychological symptoms: too burdensome or stressful; negative psychological symptoms: subjective worsening of mood; self-harm; and hospitalization) [44,47,51,53-55,59,60,62,70,71,74,76].

#### Prevalence of Negative Psychological Symptoms: Too Burdensome or Stressful

Pooled prevalence of negative psychological symptoms: too burdensome or stressful was 0.04 (95% CI 0.02-0.07; *P*<.001). Heterogeneity was high (Cochran Q_7_=26.31; *P*<.001; *I*^2^= 73.4%), suggesting low-quality evidence that the prevalence of these negative psychological symptoms was related to mood monitoring.

**Figure 4.**
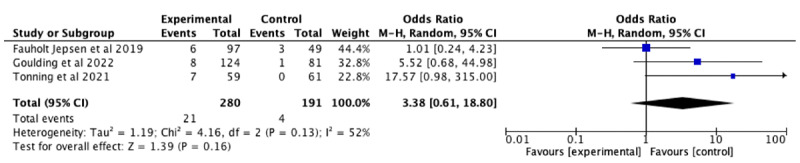
Forest plot of odds ratios of negative psychological symptoms: too burdensome or stressful adverse events in randomized controlled trials comparing mood monitoring experimental arms versus non–mood monitoring control arms [54,59,62].

We compared the difference in negative psychological symptoms: too burdensome or stressful in RCTs with an appropriate non–mood monitoring control group (Figure 4), which included 3 studies [57,59,62]. The pooled odds ratio was 3.38 (95% CI 0.61-18.80). Heterogeneity was moderate (τ²=1.19; *χ*^2_2_^=4.2; *P*=.13; *I*²=52%). There was no statistically significant difference between burdensome or stressful adverse events in mood monitoring controls and non–mood monitoring controls, but our analyses were limited by the paucity of data (*P*=.16).

#### Prevalence of Negative Psychological Symptoms: Subjective Worsening of Mood

Pooled prevalence of negative psychological symptoms: subjective worsening of mood was 0.02 (95% CI 0.01-0.02; *P*=.001). Heterogeneity was very low (Cochran Q_3_=1.77; *P*=.62; *I*^2^= 0%), providing moderate evidence of a worsening of mood related to mood monitoring in 2% of the participants; however, as these data were from only 4 (5%) of the 77 nonrandomized studies reporting a rare outcome, the evidence is not robust enough.

#### Prevalence of Self-Harm

Pooled prevalence of self-harm was 0.05 (95% CI −0.02 to 0.10; *P*<.001). Heterogeneity was very high (Cochran Q_3_=42.86; *P*<.001; *I*^2^=93%), again suggesting low-quality evidence that the prevalence of self-harm was related to mood monitoring.

#### Prevalence of Hospitalization

Pooled prevalence of hospitalization was 0.06 (95% CI 0.04-0.09; *P*=.26). Heterogeneity was low (Cochran Q_2_=2.70; *P*=.26; *I*^2^=26.1%). Despite this nonsignificant relationship between mood monitoring and the risk of hospitalization, this remains a serious adverse event, and there should be improved monitoring of hospitalization that might be associated with mood monitoring.

## Discussion

This systematic review assessed adverse events and examined acceptability and usability issues in mood monitoring and ambulatory assessment protocols. We investigated facilitators and barriers, as well as adverse effects, associated with mood monitoring among both people with depression and those with BD. Overall, the reporting of adverse events (15/77, 19%) and usability issues (20/77, 26%) was very poor, and we were unable to infer the causality of these adverse events. Furthermore, due to the severe underreporting of adverse events as well as heterogeneity and publication bias in the included studies, there was limited certainty in the prevalence, duration, and severity of these adverse events. The most frequent facilitators of mood monitoring were perceiving mood monitoring as helpful (9/24, 38%) and ease of use (10/24, 42%). The most common barriers to mood monitoring were that it was time consuming or that there were technical issues with the mood monitoring app (9/24, 38%). There were a range of suggestions for improvements, with the most common being a request for personalization of the app to the user’s needs for mood monitoring. In terms of adverse events, evidence that approximately 2% (1/77) of people with depression or BD experience a lowering of mood with mood monitoring was the most robust. Evidence that mood monitoring might be burdensome to patients or lead to self-harm was of very low quality. There was no significant association between mood monitoring and hospitalization.

While many users found mood monitoring protocols to be therapeutic, our findings also demonstrate that a small number of users in these studies reported negative psychological effects. It is unclear whether this highlights a potential area for improvement or whether the relationship is noncausal. Our quantitative findings are in keeping with other reviews of adverse events in smartphone app interventions; for example, Linardon et al [9] found a deterioration rate of 6%. In our review, only a minority of the studies (17/77, 22%) explicitly reported adverse events, and only 12 (16%) of the 77 studies explicitly reported whether a deterioration in mood had occurred. Any adverse findings are important because mood monitoring measures are more commonly used in longitudinal studies. Negative experiences could obfuscate any positive effects if mood monitoring is used as an assessment measure in interventional studies.

While we cannot demonstrate here that mood monitoring causes a worsening of mood, this study identifies some plausible ways in which mood monitoring could potentially worsen mood or anxiety and potentially increase subjective stress. These include mood monitoring serving as an unwelcome reminder of mental illness, the burden of mood monitoring, the intervention being time consuming and repetitive, and technical problems that may frustrate users. Although these mechanisms remain speculative, other research suggests that rumination, perceived helplessness, and intrusive reminders may contribute to mood deterioration [6]. It was unclear whether these feelings of worsening mood were experienced by everyone or by only certain individuals, and not all studies reported negative psychological effects. However, some concerns were reported by participants in the majority of the studies (10/15, 67%; eg, increases in subjective stress). These adverse effects must be considered carefully when determining who may or may not benefit from these interventions moving forward. It may be necessary to incorporate additional therapeutic elements into the app to offset the negative effects or offer additional support to users.

By reviewing acceptability and usability issues of mood monitoring and ambulatory assessment protocols, we demonstrate the great potential to improve existing protocols to maximize acceptability, engagement, adherence, user experience, usability, and safety. The data on suggested improvements were limited, with only 7 (9%) of the 77 studies reporting data, and included personalization (3/7, 43%) and additional human contact (2/7, 29%). Other research that has explored this in more detail concluded that users place high emphasis on personalization and customization and continue to greatly value human contact with their clinician—the tool is an aid to this contact, not a replacement for it [6]. Other research has suggested that personalization should be a core feature of any future protocol development to maximize successful implementation and uptake [6]. Qualitative research demonstrates that users desire an intuitive and easy-to-use passive data protocol that builds on their existing strengths, with high emphasis on personalization and customizability [6]. Such tools may allow users to self-manage their mood disorder in their own way while retaining control over their data. Reported types of personalization include data collection, data sharing, feedback provided, and methods of interaction with the protocol (eg, notifications or wearables) [6].

In terms of the limitations of the review, only a small proportion of studies provided data, and there was evidence of publication bias in reporting as well as high heterogeneity in some of the outcomes. Many studies were nonrandomized or observational with small sample sizes or few observation weeks. As some adverse events were rare, attribution to mood monitoring rather than another factor, such as concurrent treatment or natural history of the condition, requires caution, especially in relation to any inference of causality or precision in estimates of incidence. The review combined multiple heterogeneous studies, and while this is a common limitation of many systematic reviews, the heterogeneity in some analyses was substantial, decreasing certainty in the result of the analyses. We could not ascertain the causes of heterogeneity, but it may be due to the wide range of mood monitoring and assessment protocols used across the included studies. Given the limited number of high-quality RCTs comparing mood monitoring with appropriate controls, causal inferences could not be drawn. Moreover, as is typical when assessing adverse events, issues of attribution and relatedness limit causal interpretation [32].

The level of detail provided in these studies was limited, and it was difficult to establish whether the adverse effect was related to the intervention itself. Furthermore, many studies included a variety of features beyond mood monitoring, which made it challenging to isolate the specific negative effects of mood monitoring. There were limited data on the contextual factors that might influence participants’ experiences of mood monitoring in depression. These factors include differences between digital and nondigital formats; wearable and nonwearable devices; active and passive data collection; EMA and non-EMA designs; interventions with therapeutic elements and those without; and human-, avatar-, and digitally supported interventions. Participant-related factors such as country income level, age, depression severity, ethnicity, gender, socioeconomic status, and disability were also underrepresented [6]. Nonetheless, this review focuses on actual intended use based on empirical data, often over prolonged periods of time, rather than focusing on hypothetical use derived from focus groups.

However, there are grounds to think that harm, safety, and usability are not given the priority expected for devices used in people with serious mental illness and that published reports should systematically report adverse events and usability to ensure that both these issues can be monitored and addressed. Future protocols should measure potential mood deterioration objectively to enable these quantitative data to be triangulated with qualitative data, allowing any potential causality to be inferred or refuted. Low rates of reporting negative effects are common across digital interventions and may be hindering the development of safer interventions [9]. It may be possible for major funders (eg, Wellcome Trust and UK Research and Innovation) and regulatory bodies to introduce the requirement for grant holders and developers of technology to use responsible innovation networks that explicitly ask research teams to consider negative adverse effects and their measurement, mitigation, and reporting (eg, internationally recognized standards such as Good Clinical Practice [86]). The data in this review show that previous studies have not considered these issues adequately.

## Supplementary material

10.2196/79500 Multimedia Appendix 1 Supplementary figures, tables, and materials.

10.2196/79500 Checklist 1 PRISMA checklist.

## Abbreviations

BD: bipolar disorder
EMA: ecological momentary assessment
PRISMA: Preferred Reporting Items for Systematic Reviews and Meta-Analyses
RCT: randomized controlled trial
RoB 2: Cochrane risk-of-bias tool
ROBINS-I: Cochrane risk of bias in nonrandomized studies of interventions tool
