# Supplementary material for: Adverse Events of Mood Monitoring and Ambulatory Assessment in Depression and Bipolar Disorder: Systematic Review and Meta-Analysis
Source: JMIR Ment Health. 2025 Oct 23;12:e79500. doi: 10.2196/79500 (PMC12548826; doi:10.2196/79500)

Multimedia Appendix 1

**Supplementary Table 1: Characteristics of included studies**

| **Study** | **Country** | **n** | **Mean age in years (SD)** | **% female** | **Study Design** | **Mood disorder** | **Intervention** | **Ambulatory assessment/mood tracking  Procedure** | **Ambulatory assessment duration** |
| --- | --- | --- | --- | --- | --- | --- | --- | --- | --- |
| Anzy et al 2021 | Czech Republic | 99 | 37.1 (11) | 61 | Non-randomised | Bipolar Disorder | Aktibipo self-rating questionnaire | ASERT mood self-reports weekly - 10 items that map depressive, manic and nonspecific symptoms on a likert scale. | 18 months |
| Hidalgo-Mazzei et al 2016 | Spain | 51 | 43.92 (11.36) | 42.9 | Non-randomised | Bipolar Disorder | SIMPLe 1.0 - Self-report 5 item test assessing mood, energy, sleep duration, medication adherence and irritability - daily. DSM-5 criteria for manic/depressive episodes - weekly. Daily notification of psychoeducation relapse prevention message (500 different messages possible) targeting specific situations based on ambulatory assessment data. | Self-report 5 item test assessing mood, energy, sleep duration, medication adherence and irritability - daily. DSM-5 criteria for manic/depressive episodes - weekly. | 3 months |
| Hidalgo-Mazzei et al 2018 | Spain, Argentina | 201 | 36.59 (11) | 63.2 | Non-randomised | Bipolar Disorder | SIMPLe 1.5 - Self-report 5 item test assessing mood, energy, sleep duration, medication adherence and irritability - daily. DSM-5 criteria for manic/depressive episodes - weekly. Daily notification of psychoeducation relapse prevention message (500 different messages possible) targeting specific situations based on ambulatory assessment data. Additional app components/modules: medication reminders, personalised prodromal symptoms, gamification module, mood-chart sharing, psychoeducational messages community. | Self-report 5 item test assessing mood, energy, sleep duration, medication adherence and irritability - daily. DSM-5 criteria for manic/depressive episodes - weekly. | 12 months |
| Garcia-Estela et al 2022 | Spain, Chile, Argentina, Mexico, Colombia, Guatemala, Brazil, Other | 503 | 34.74 (10.48) | 67.7 | Non-randomised | Bipolar Disorder | SIMPLe 1.5 - Self-report 5 item test assessing mood, energy, sleep duration, medication adherence and irritability - daily. DSM-5 criteria for manic/depressive episodes - weekly. Daily notification of psychoeducation relapse prevention message (500 different messages possible) targeting specific situations. Additional app components/modules: medication reminders, personalised prodromal symptoms, gamification module, mood-chart sharing, psychoeducational messages community. | Self-report 5 item test assessing mood, energy, sleep duration, medication adherence and irritability - daily. DSM-5 criteria for manic/depressive episodes - weekly. | 6 months |
| Bauer et al 2023 | Germany, USA | 609 | 40.3 (11.8) | 71.4 | Non-randomised | Bipolar Disorder | ChronoRecord - daily mood via 100 point VAS, sleep, life events, menstrual data, psychiatric medication, weekly - weight. | ChronoRecord - daily mood via 100 point VAS, sleep, life events, menstrual data, psychiatric medication, weekly - weight. | Average follow up: 227 days |
| Bos et al 2022 | The Netherlands | 20 | 20-35 years: n = 9, 36-50 years: n = 8, 51-65 years: n = 3 | 80 | Non-randomised | Bipolar Disorder | 5x EMA smartphone assessments daily - 29 items assessing monetary mood, symptoms, sleep and activities. Weekly ASRM, QIDS-SR-16 delivered via RoQua platform. | 5x EMA smartphone assessments daily - 29 items assessing monetary mood, symptoms, sleep and activities. Weekly ASRM, QIDS-SR-16 delivered via RoQua platform. | 4 months (range: 16-32 weeks) |
| Bowden et al 2021 | USA | 20 | Not reported | Not reported | Non-randomised | Bipolar Disorder | KIOS app - self report assessment of 8 different symptoms e.g sadness/pessimism and delivery of guidance in relation to symptom change. | KIOS app - self report assessment of 8 different symptoms e.g sadness/pessimism and delivery of guidance in relation to symptom change. | 3 months |
| Dominiak et al 2022 | Poland | 84 | 36.2 (9.5) | 55 | Non-randomised | Bipolar Disorder | BDmon app - Passive ambulatory assessment: phone/SMS logs, participant speech information extracted from daily phone calls, Active ambulatory assessment: self report mood | BDmon app - Passive ambulatory assessment: phone/SMS logs, participant speech information extracted from daily phone calls, Active ambulatory assessment: self report mood | mean: 208 days (SD: 32) |
| Emden et al 2021 | Germany | Total: 997, Depression: 409, Bipolar Disorder: 48, Anxiety: 58, Psychosis: 21, Healthy controls: 458 | 35.99 (13.57) | 67.3 | Non-randomised | Bipolar Disorder | ReMAP system | ReMAP system - Active ambulatory assessment - single mood likert scale, single item sleep scale assessing self-report sleep time, voice sample - weekly. Passive ambulatory assessment: step-count, GPS location, accelerometer - continuous. | 12 months |
| Stanislaus et al 2020 | Denmark | Bipolar Disorder: 203, Unaffected first-degree relatives: 54, HC: 109 | Median: 28 (IQR: 24-35) | 69 | Non-randomised | Bipolar Disorder | Monsenso system | Monsenso system - Active ambulatory assessment: daily smartphone self monitoring items - mood and activity level, HDRS-17 & YMRS every 3 days. Passive ambulatory assessment: Objective smartphone data - phone usage, call/SMS logs, step count | Median 106 days (IQR: 48-204) |
| Lee et al 2022 | South Korea | Total: 495, Depression: 95, Bipolar Disorder: 175 | Subjects included in analysis: 23.3 (3.63), subjects not included in analysis: 22.8 (3.10) | Subjects included in analysis: 54.4, subjects not included in analysis: 59.1 | Non-randomised | Bipolar Disorder | eMoodChart system | eMoodChart system - Active ambulatory assessment: self report daily mood and energy. Passive ambulatory assessment: Fitbit measuring step-count, heart rate, sleep, ambient light (android online) | mean: 279.7 days (SD: 263.5), median: 505 days (range: 72-1515) |
| Born et al 2014 | Germany | 108 | 39.3 (13.3) | 47.2 | Non-randomised | Bipolar Disorder | NIMH Life Chart Methodology - prospective | NIMH Life Chart Methodology - prospective —twice daily mood self-rating | Unclear - potentially 3 years |
| Lieberman et al 2011 | USA | 64 | Not reported | Not reported | Non-randomised | Bipolar Disorder | MoodChart | MoodChart - daily self report mood via email/online, Social Rhythm Metric - activity level over the previous 7 days | mean: 84 (range: 42-90) |
| Kupka et al 2005 | USA, Netherlands, Germany | 539 | 42.1 (11.5) | 56 | Non-randomised | Bipolar Disorder | NIMH Life Chart Methodology - prospective | NIMH Life Chart Methodology - prospective — daily mood self-rating | 1 year |
| O’Rourke et al 2021 | Canada, USA, UK, South Africa, Australia | 50 | 50% >45 years | Not reported | Non-randomised | Bipolar Disorder | Twice daily Bipolar Disorder Symptom Scale | Twice daily Bipolar Disorder Symptom Scale | 4+ months |
| Tseng et al 2022 | Taiwan | 159 | 34.5 (11.34) | 55.97 | Non-randomised | Bipolar Disorder | Smartphone app collecting daily/weekly active and passive ambulatory assessment. | Active ambulatory assessment: daily mood, sleep duration. Weekly ASRM, DASS-21. Passive ambulatory assessment: GPS location | number of days on which participants performed self-assessments - mean: 94.25 days (median 52.5, range 2 to 398) |
| Ebner-Priemer et al 2020 | Germany | 31 | 44 (11.9) | 55 | Non-randomised | Bipolar Disorder | MovisensXS | MovisensXS system - Active ambulatory assessment: self-report mood, sleep diary.  Passive ambulatory assessment: call/text logs, GPS data, velocity, step-count. | 12 months |
| Gideon et al 2016 | USA | 37 | Not reported | Not reported | Non-randomised | Bipolar Disorder | PRIORI app | PRIORI system - records speech made on telephone calls. Weekly HDRS, YMRS. | 6-12 months. Mean: 29.2 weeks (SD: 16.4) |
| Schneider et al 2022 | Czech Republic | Bipolar disorder: 35, HC: 26 | 39.75 (SD: 12.85) | 60 | Non-randomised | Bipolar Disorder | MINDPAX - actigraphy | MINDPAX - actigraphy measuring sleep data | 3 months |
| Scharer et al 2015 | Germany | 54 | 40.6 | 46.3 | Non-randomised | Bipolar Disorder | PLC app | PLC app - daily self report mood | 18 months |
| van den Heuvel et al 2018 | Netherlands | 66 | 45.17 (10.67) | 66.7 | Non-randomised | Bipolar Disorder | PHR-BD system | PHR-BD system - including 9 modules covering: medical record, medication, treatment and medical passport, general information about BD, medical results/reports, platform to send messages to appointed clinician, mood chart with daily self report mood, personal crisis plan. | 12 months |
| Arribas et al 2018 | UK | 139 | Bipolar Disorder: 38 (+/-21), Borderline Personality Disorder: 34 (+/-15), HC:37 (+/-20) | Bipolar Disorder: 69.8%, Borderline Personality Disorder: 94%, HC: 66% | Non-randomised | Bipolar Disorder | AMoSS study system | AMoSS study system - Active ambulatory assessment: daily mood rating across categories of anxiety, elation, sadness, anger, irritability and energy using MoodZoom questionnaire, ASRM, QIDS-SR16, EQ-5D, GAD-7 assessed weekly. Passive ambulatory assessment: GPS, actigraphy, ambient light, call/SMS logs, heart rate via smartphone/Fitbit/GENEActive accelerometer/Proteus patch (heart rate data only for one week). | 3 months, with 61 participants continuing for 12 months |
| Lewis et al 2023 | UK | 649 | 53 (range: 22-83) | 68 | Non-randomised | Bipolar Disorder | Bipolar Disorder Research Network using True Colours | Bipolar Disorder Research Network using True Colours: weekly ASRM/QIDS-16SR | 21 months |
| McKnight et al 2017 | UK | 367 | 41 (SD: 13.7, range: 16-76) | 66.7 | Non-randomised | Bipolar Disorder | OXTET-1 using True Colours | OXTET-1 using True Colours - ASRM/QIDS-SR16 delivered via weekly SMS/email | 27.5 ± 22.5 months (range: 1-81) |
| Ortiz et al 2023 | Canada | 87 | 38.9 (SD: 12.4) | 67.8 | Non-randomised | Bipolar Disorder | E-monitoring system | E-monitoring system - Active ambulatory assessment: daily rating of mood, anxiety, energy level using e-VAS. Weekly: PHQ-9, ASRM. Passive ambulatory assessment: Oura Health Oy 3d accelerometer/hyroscope measuring activity, sleep, Infrared optical pulse measuring heart rate, heart rate variability | 229.4 days (± 12.4) |
| Aguilera et al 2017 | USA | 91 | Text message group: 51.71 (11.55), Control group: 51.83 (11.73) | 78.8 | Non-randomised | Depression | Group CBT with text messaging intervention (HealthySMS), Group CBT without text messaging intervention | HealthySMS: up to 5 automated text messages including: daily mood rating, supplementation of therapy content, weekly reminders to attend CBT sessions, monthly opt out messages to end message delivery if desired. Weekly PHQ-9 for 16 weeks. | median: 13.5 weeks |
| Aikens et al 2015 | USA | 221 | 51.4 (12.7) | 78.6 | Non-randomised | Depression | Automated Interactive Voice Response telephone calls | Automated Interactive Voice Response telephone calls assessing symptom severity - PHQ-9 and antidepressant adherence. | 6 months |
| Benedyk et al 2023 | Germany | Total: 65, Depression: 24, Schizophrenia: 20, Healthy Controls: 21 | Schizophrenia group: 34.6 (10.3), Depression group: 38.0 (13.5), Healthy controls: 38.5 (11.1) | Schizophrenia group: 75%, Depression group: 62.5%, Healthy controls: 48% | Non-randomised | Depression | Smartphone-based self-ratings of wellbeing, social context and anxiety level, step counter, real-life GPS location tracking)  and psychological inventories | INDICATE-N/Movisens GmbH system. Active ambulatory assessment: e-diary: twice daily fixed time smartphone-based self-ratings of mood, affect and social context via 21 e-diary items. Mood assessed via six item Multidimensional Mood Questionnaire. Participants also completed 3 intense EMA phases with 6 daily prompts for 10 days each.                                   Passive ambulatory assessment: Step count, real-life GPS location tracking. | 6 months |
| Bonilla-Escribano et al 2023 | Spain/France | Total: 275, PTSD: 74, Depression: 40, Binge Eating Disorder: 23, Agoraphobia: 18, AUD: 9, Bipolar Disorder: 6, OCD: 7, Panic Disorder: 26, Social Anxiety Disorder: 4, SUD (non-alcohol): 5 | 40 (14) | 67.27 | Non-randomised | Depression | Memind & EB2 apps | Memind app: 4 question daily ambulatory assessment inspired by the Salzburg Suicide Process Questionnaire. 4 questions pulled from a 32 item pool. | 6 months |
| Carpenter et al 2021 | USA | 20 | 47.1 | 80 | Non-randomised | Depression | Maintenance TMS | Weekly PHQ-9 delivered via app | 12 months |
| de Angel et al 2023 (80) | UK | 66 | 34.6 (11.1) | 61 | Non-randomised | Depression | Psychotherapy | RADAR-base system PHQ-8, RSES, speech task – weekly. Heartrate, step-count, GPS location, acceleration, light levels, phone interaction, nearby Bluetooth device detection, battery level, weather, sleep, app usage metrics – continuous via Fitbit/RADAR-base app. | 7 months |
| Drake et al 2013 | UK | 20 | Completers: 38.9 (12.6), Dropouts: 32.8 (15.3) | 75 | Non-randomised | Depression | Moodscope website | PHQ-9, GAD-7 - weekly. Moodscape self-rated mood - selecting which of each 20 interactive mood-adjective playing cards describes current mood. | 3 months |
| Funkhouser et al 2024 | USA | 90 | 16.57 (1.43) | 63 | Non-randomised | Depression | EARS app | EARS app - Active: daily mood rating. Passive: keyboard inputs. | 12 months |
| Helmich et al 2022 | Netherlands | 41 | 40.1 (14.4) | 85 | Non-randomised | Depression | TRANS-ID Recovery System | TRANS-ID Recovery System - 27 item EMA delivered 5 times a day about current feelings, activities and surroundings incorporating VAS. Weekly 14 item depressive symptom scale of SCL-90. Weekly rating of how much depressive symptoms have bothered them. | 4-6 months |
| Janevic et al 2016 | USA | 32 | 58 (13) | 78 | Non-randomised | Depression | IVR | Automated Interactive Voice Response telephone calls assessing symptom severity - 6 item version of the 17 item HAM-D, PGI, WSA - and offering tailored treatment recommendations and individualised feedback. | 14w |
| Kathan et al 2022 | Germany | 48 | Not reported | Not reported | Non-randomised | Depression | MAIKI system | MAIKI system - Passive ambulatory assessment: app sessions and app useage, metadata on general phone settings and phone actions, GPS data, communication information e.g contacts, call and SMS data. Active ambulatory assessment - GAD-7, PSS, PHQ-9 - weekly, items extracted from the CESD, PDD, PSQI, PHQ-2 - daily | 3 months |
| Kline et al 2024 | USA | 207 | 38.74 (10.42) | 81.6 | Non-randomised | Depression | MLife app | MLife app. Active ambulatory assessment: 3 times daily modified PHQ-9 assessing symptoms over previous 4 hours, VAS of mood, optional diary entry. | 3 months |
| Matcham et al 2022 | UK, Spain, Netherlands | 623 | 46.4 (15.3) | 75.6 | Non-randomised | Depression | RADAR-base system | RADAR-base system PHQ-8, RSES, speech task – weekly. Heartrate, step-count, GPS location, acceleration, light levels, phone interaction, nearby Bluetooth device detection, battery level, weather, sleep, app usage metrics – continuous via Fitbit/RADAR-base app. | 2 years |
| Mcintyre et al 2021 | Canada | 523 | 46 (12.7) | ~76 exact numbers not reported | Non-randomised | Depression | Mind.me system | Mind.me system - Passive ambulatory assessment: daily call/SMS count, location variance, normalised entropy, number of GPS geolocation coordinate clusters, total distance (km), mean absolute deviation in distance (km). | 3 months |
| Meyerhoff et al 2021 | USA | 282 | 38.9 (11.9) | 79.1 | Non-randomised | Depression | Passive Data Kit system | Passive Data Kit system - Passive ambulatory assessment: GPS coordinates, phone/SMS logs, duration/length, open foreground apps. | 4 months |
| Osgood-Hynes et al 1998 | USA | 41 | 42 (13) | 71 | Non-randomised | Depression | COPE self-help system and Interactive Voice Response telephone calls | Automated Interactive Voice Response telephone calls assessing symptom severity - 6 item version of the 17 item HAM-D, PGI, WSA - and offering tailored treatment recommendations and individualised feedback. | 3 months |
| Sharp et al 2020 | USA | 28 | PA pilot: 47.4 (12.61), BC pilot: 56.4 (8.10) | PA pilot: 78.57, BC pilot: 100 | Non-randomised | Depression | Active Living Evday Day counselling programme - 12 weekly group educational sessions about ways to increase physical activity with Fitbit/Actigraph 3GTX+ monitoring activity levels | Fitbit/Actigraph 3GTX+ monitoring activity levels | 3 months |
| Smit et al 2023 | Netherlands | 56 | 45.9 (12.9) | 83.9 | Non-randomised | Depression | 5x measurements per day assessing restlessness and mood for 4 months with reminder sent via text-message. SCL-90 weekly for 6 months | 5x measurements per day assessing restlessness and mood for 4 months with reminder sent via text-message. SCL-90 weekly for 6 months | 5x daily assessments - 4 months, SCL-90 weekly for 6 months |
| Vachon et al 2016 | France | 30 | 51.4 (9.6) | 62.5 | Non-randomised | Depression | 2x daily self-report mood | 2x daily self-report mood assessing affective/cognitive states relating to depression, visual analogue scale for depression, self-esteem, physical self, quality of life, coping, rumination, anxiety. | 5 months |
| Van der Watt et al 2022 | South Africa | 50 (30 with Depression, 11 with Bipolar Disprder, 9 with diagnosis: Other) | 39.49  (11.17) | 74 | Non-randomised | Depression | Weekly QIDS, ASRM administered via telephone | Weekly QIDS, ASRM administered via telephone | 4 months |
| Webb et al 2022 | USA | Total: 39, Adolescents with depression: 24 | 15.7 (1.9) | 66.7 | Non-randomised | Depression | 12 weekly course of Behavioural Activation, 2x 5 day ambulatory assessment periods measuring affect, probabilistic reward tasks, fMRI reward tasks | Weekly SHAPS, CES-D, BADS-SF for 15 weeks | 15w |
| Xia et al 2022 | USA | 41 (24 with depression) | 23.5 (3.5) | 68% | Non-randomised | Depression | Beiwe system - GPS geolocation and accelerometer data | Beiwe system - GPS geolocation and accelerometer data - continuous | 3 months |
| Lee et al 2022 | South Korea | Total: 495, MDD: 95, BD: 175 | Subjects included in analysis: 23.3 (3.63), subjects not included in analysis: 22.8 (3.10) | Subjects included in analysis: 54.4, subjects not included in analysis: 59.1 | Non-randomised | Depression | eMoodChart system | eMoodChart system - Active ambulatory assessment: self report daily mood and energy. Passive ambulatory assessment: Fitbit measuring step-count, heart rate, sleep, ambient light (android online) | mean: 279.7 days (SD: 263.5), median: 505 days (range: 72-1515) |
| Bilderbeck et al 2016 | UK | 121 | 44 (1) | 72.7 | RCT | Bipolar Disorder | Intervention: Facilitated Integrated Mood Management (therapist administered psychoeducation). Comparator: Manualised Integrated Mood Management (self-administered psychoeducation) | QIDS-SR-16, ASRM - administered weekly via TrueColours | 12 months |
| Denicoff et al 2002 | USA | 52 | 41.3 (11.4) | 52 | RCT | Bipolar Disorder | Intervention: year 1: lithium, year 2: switch to carbamazepine, year 3: lithium & carbamazepine. Comparator: year 1: carbamazepine, year 2: switch to lithium, year 3: lithium & carbamazepine | NIMH Life Chart Methodology - prospective —twice daily mood self-rating | 3 years |
| Faurholt-Jepsen et al 2015 | Denmark | 67 | 29.3 (8.43) | 67 | RCT | Bipolar Disorder | Intervention: MONARCA system plus: 1. study nurse reviewing data and contacting patients if sign of deterioration to offer advice 2. self-monitored data graphically visualised. Comparator: Normal smartphone use | Daily smartphone self monitoring - mood, sleep duration, medication taken, activity, irritability, mixed mood, cognitive problems, alcohol consumption, stress, menstruation, individualised EWS | 6 months |
| Faurholt-Jepsen et al 2019 | Denmark | 129 | 43 (12) | 59 | RCT | Bipolar Disorder | Intervention: Monsenso system plus: 1. study nurse reviewing data and contacting patients if sign of deterioration to offer advice 2. self-monitored data graphically visualised. Comparator: Normal smartphone use | Daily smartphone self monitoring items - mood, sleep duration, medication taken, activity, irritability, mixed mood, cognitive problems, alcohol consumption, stress, menstruation, individualised EWS, anxiety, self-defined personal parameters, free-text note.                     Objective smartphone data - phone usage, social activity, step count, GPS location | 9 months |
| Faurholt-Jepsen et al 2020 | Denmark | 98 | 42.69 (13.46) | 52 | RCT | Bipolar Disorder | Intervention: Monsenso system plus: 1. study nurse reviewing data and contacting patients if sign of deterioration to offer advice 2. self-monitored data graphically visualised. Comparator: Usual care | Daily smartphone self monitoring items - mood, sleep duration, medication taken, activity, irritability, mixed mood, cognitive problems, alcohol consumption, stress, menstruation, individualised EWS, anxiety, self-defined personal parameters, free-text note.                     Objective smartphone data - phone usage, social activity, step count, GPS location | 6 months |
| Gliddon et al. 2018 | Austrailia & USA | 304 | 39.47 (11.19) | 82 | RCT | Bipolar Disorder | Intervention 1: Discussion forum plus MoodSwings-Plus: MoodSwngs plus additional CBT-based interactive elements – tools to support mood and medication monitoring, life-chart development, cognitive strategies, motivational interviewing techniques, self reflection, problem solving, identification of personal triggers and a relapse prevention plan.    Intervention 2: Discussion forum plus MoodSwings: Online intervention comprising: mood monitoring, assessing prodromal mood states, preventing relapse, setting SMART goals. Online delivery of MAPS (Mood Assessment Prevent SMART) programme.      Comparator:  Discussion forum | Online mood-monitoring via MoodSwings & MoodSwings-Plus websites | 12 months |
| Lauder et al 2015 | Australia | 156 | MoodSwings-Plus: 39.87 (11.26), MoodSwings: 41.35 (9.85) | 62 | RCT | Bipolar Disorder | Intervention: MoodSwings-Plus: MoodSwings plus additional CBT-based interactive elements – tools to support mood and medication monitoring, life-chart development, cognitive strategies, motivational interviewing techniques, self reflection, problem solving, identification of personal triggers and a relapse prevention plan. Comparator: MoodSwings: Online intervention comprising: mood monitoring, assessing prodromal mood states, preventing relapse, setting SMART goals. Online delivery of MAPS (Mood Assessment Prevent SMART) programme. | Online mood-monitoring via MoodSwings & MoodSwings-Plus websites | 12 months |
| Castle et al 2018 | Australia | 84 | Control group: 42.6 (11.3), Treatment group: 41.6 (11.0) | 84 | RCT | Bipolar Disorder | Intervention: Structured group programme comprising an initial block of 12 weekly sessions with 3 additional monthly booster sessions to support participants in applying knowledge and skills to their lives. Included weekly telephone calls to remind participants of the next group session and to offer support for homework tasks. Comparator: Usual care plus weekly telephone calls | Weekly telephone calls – weekly for 12 weeks. | 12 months |
| Goulding et al 2022 | USA | 205 | 42 (12) | 61 | RCT | Bipolar Disorder | Intervention: Livewell, Comparator: Usual care | Smartphone based self management intervention - daily and weekly check-ins for weeks 1-16. Daily - adherence, sleep, duration, routine, wellness levels. Weekly - symptom severity scoring for all individual DSM-IV mood symptoms. | 4 months |
| Petzold et al. 2014 | Germany | 73 | Intervention: 44.32 (11.63), Control: 42.69 (12.34) | 45 | RCT | Bipolar Disorder | Intervention: 6 weekly group Psychoeducation sessions plus 54 weeks of ChronoRecord. Comparator: 6 weekly supportive non-structured group meetings plus 54 weeks of daily unstructured computer-based self-reports/diary | ChronoRecord - daily mood, sleep, life events, menstrual data, psychiatric medication, weekly – weight. | 12.5 months |
| Van den Berg et al 2023 | Netherlands | 62 | Intervention: 46.5 (11.1), Control: 42.73 (13.0) | 58 | RCT | Bipolar Disorder | Intervention: Imagery Focussed CBT. Comparator: Group Psychoeducation | NIMH Life Chart Methodology - daily mood and anxiety self-rating. | 4 months |
| Goldberg et al. 2006 | USA | 177 | Intervention: 38.5 (9.1), Control 37.7 (10.5) | 56.5 | RCT | Bipolar Disorder | Intervention: Lamotrogine monotherapy. Comparator: placebo | NIMH Life Chart Methodology - daily mood self-rating | 6.5 months |
| Langosch et al 2008 | Germany | 44 | Quetiapine: 45.4 (11), Valproate 37.8 (13.8) | 60 | RCT | Bipolar Disorder | Intervention: Quetiapine monotherapy. Comparator: Valproate monotherapy | NIMH Life Chart Methodology - daily mood self-rating | 12 months |
| Leverich et al 2006 | USA | 159 | 41.6 (12.2) | 47.8 | RCT | Bipolar Disorder | Intervention/Comparator: Buproprion or sertraline or venlafaxine as an adjunct to mood stabilisers | NIMH Life Chart Methodology - daily mood self-rating | 12 months |
| Lieberman et al 2010 | USA | 48 | Paper chart: 39.5 (12.9), Online chart: 35.8 (12.0) | 75 | RCT | Bipolar Disorder | Intervention: NIMH Life Chart Methodology - prospective — daily mood self-rating. Comparator: Online Life Chart adaptation | NIMH Life Chart Methodology - prospective — daily mood self-rating / online Life Chart adaptation | 3 months |
| Depp et al 2012 | USA | 56 | Paper chart: 46.1 (13.5), Phone chart: 44.0 (14.0) | 57.5 | RCT | Bipolar Disorder | Intervention: Paper and pen life charting. Comparator: Smartphone based life charting | NIMH Life Chart Methodology - prospective — daily mood self-rating via paper and pen and via smartphone | 3 months |
| Pahwa et al 2023 | USA | 122 | 43.75 (14.05) | 68.60 | RCT | Bipolar Disorder | Intervention: KIOS app. Comparator: eMoods app. | KIOS app - self report assessment of 8 different symptoms e.g sadness/pessimism and delivery of guidance in relation to symptom change.  eMoods app – self report mood and symptoms diary tracking daily outlook, motivation, habits, sleep, medications etc | 12 months |
| Aikens et al 2022 | USA | 204 | 49 | 81 | RCT | Depression | Intervention: Automated Interactive Voice Response telephone calls. Comparator: Enhanced usual care - usual care plus printed self-management material at baseline and assigned family/friend to discuss this with weekly | Automated Interactive Voice Response telephone calls assessing symptom severity - PHQ-9 and antidepressant adherence. | 12 months |
| van Genugten et al. 2021 | Netherlands | 943 | 37.3 (13.2) | 66 | RCT | Depression | Intervention: bCBT: psychoeducation, behavioural activation, cognitive restructuring, relapse prevention. Comparator: TAU | Daily self-monitoring of mood state via VAS, cognitions, activities, social interaction, and sleep via a smartphone app. Ambulatory assessment protocol varied over course of the study, one some days 3x daily mood ratings. | 12w |
| Arean et al 2016 | USA | 1110 | 18-30: 584 (53.6%), 31-40: 243 (22.4%), 41-50: 151 (13.9%), 51-60: 84 (7.7%), 61-70: 20 (1.8%), 71+: 6 (0.6%) | 78.4 | RCT | Depression | Intervention arm 1: iPST - an app based on problem solving therapy, Intervention arm 2: Project Evo - a therapeutic video game meant to improve cognitive skills associated with depression, Intervention arm 3: Health Tips - an app that provides information about strategies to improve mood. | Active ambulatory assessment: PHQ-9, SDS, Global Impression of Change Scale, 3 question sleep assessment, Mental Health Services Used - weekly. PHQ-2 - daily. Passive ambulatory assessment - time of call/SMS, call duration, SMS length, activity type, distance travelled. | 12w |
| Pratap et al 2018 | USA | 1083 | 18-30: 456 (43.1%), 31-40: 320 (30.2%), 41-50: 199 (18.8%), 51-60: 63 (6.0%), 61-70: 18 (1.7%), 71+: 2 (0.2%) | 70.9 | RCT | Depression | Intervention arm 1: iPST - an app based on problem solving therapy, Intervention arm 2: Project Evo - a therapeutic video game meant to improve cognitive skills associated with depression, Intervention arm 3: Health Tips - an app that provides information about strategies to improve mood. | Active ambulatory assessment: PHQ-9, SDS, Global Impression of Change Scale, 3 question sleep assessment, Mental Health Services Used - weekly. PHQ-2 - daily. Passive ambulatory assessment - time of call/SMS, call duration, SMS length, activity type, distance travelled. | 12w |
| Dai et al 2022 | USA | 106 | Intervention: 46.86 (12.1), Control: 47.47 (12.88) | Intervention: 76.2, Control: 76.6 | RCT | Depression | Intervention: I-CARE2: Integrated Coaching for Better Mood and Weight. Includes PEARLS program for collaborative stepped depression care, which uses PST augmented with behavioural activation. Comparator: TAU. In addition they receive a summary of behavioural health and weight management services at UI health and a Fitbit. | Wearable activity tracker (Fitbit) measuring sedentary minutes, lightly active minutes, minutes of heart rate zone in fat-burn, minutes of heart rate zone in cardio, total walking distance, activity calories, minutes awake in main sleep, restless count in main sleep, efficiency in main sleep, time in bed of main sleep. | 6 months |
| Tonning et al 2021 | Denmark | 120 | Intervention: 44.5 (14.0), Control: 43.4 (14.3) | Intervention: 47.5 (28), Control: 43.4 (14.3) | RCT | Depression | Intervention: Monsenso system plus: 1. study nurse reviewing data and contacting patients if sign of deterioration to offer advice 2. self-monitored data graphically visualised 3. smartphone based CBT modules. Comparator: TAU | Daily smartphone self monitoring items - mood, sleep duration, medication taken, activity, irritability, mixed mood, cognitive problems, alcohol consumption, stress, menstruation, individualised EWS, anxiety, self-defined personal parameters, free-text note.                     Objective smartphone data - phone usage, social activity, step count, GPS location | 6 months |
| Frank et al 2022 | USA | 133 | 32.94 (11.39) | 73.68 | RCT | Depression | Intervention: Passive behaviour monitoring, 10 psychoeducation modules, Cue system providing personalised micro-interventions delivered every 2/3 days, self-report completion. Comparator: Passive behaviour monitoring, self-report completion | Daily VAS rating of mood | 16w |
| Hunkeler et al 2012 | USA | 103 | Intervention: 48.49 (12.83), Usual care: 51.88 (10.56) | 79.6 | RCT | Depression | Intervention: eCare for Moods - website offering personalised self-monitoring, messaging with eCare manager, depression psychoeducation, CBT modules, online discussion group, problem-specific advice, personal database, task lists, appointment calendar. Comparator: TAU | Personalised self-monitoring via eCare for Moods - tracking health-related disability, medication adherence, side effects, alcohol and drug use, new symptoms, early warning signs. Graphs of monitoring data displayed over time. | 12 months |
| Klein et al 2016 | Germany | 1013 | Intervention: 42.8 (11.0), Control: 42.9 (11.0) | 69 | RCT | Depression | Intervention: Deprexis self-help program. Comparator: TAU | Weekly contact by trained email supporter - short feedback based on participants’ program usage over the past week. Feedback is relatively generic in nature and does not apply therapeutic strategies in detail. PHQ-9 biweekly. | 12w |
| Tuvey et al 2023 | USA | 53 | SM: 40.11 (10.65), SM + Coach: 47.5 (14.26), AC: 49.55 (14.74) | SM: 76, SM + Coach: 77, AC: 61 | RCT | Depression | Intervention arm 1: Annie Secure Messaging (SM) system, Intervention arm 2: Annie SM system with coaching | Intervention arm 1: Annie Secure Messaging (SM) system – promoting antidepressant adherence with texts around efficiacy and AEs, Intervention arm 2: Annie SM system with coaching – weekly coaching call with a study member to review weekly data reported via Annie looking at trends over time – participants asked to reflect on trends and identify any self-management issues | 12w |
| White et al 2023 | UK | 100 | 53.3 (14.3) | 76 | RCT | Depression | Intervention arm 1: RADAR-base app plus addition in-app components grounded in behavioural theory and using the Capability, Opportunity and Motivation framework of behaviour change. The app used symptom tracking, behavioural feedback via progess visualisation and instant access to researcher contact details.                               Intervention arm 2: RADAR-base app | PHQ-8, RSES, speech task – weekly. Heartrate, step-count, GPS location, app usage metrics – continuous via Fitbit/RADAR-base app | 12w |
| **ReMAP - Remote Monitoring in Psychiatry, ASERT - Aktibipo Self-rating Questionnaire, FAST - Functional Assessment Screening Tool, EMA - Ecological Momentary Assessment, DSM-5 - Diagnostic and Statistical Manual of Mental Disorders 5th edition, HC - Healthy Controls, NIMH - National Institute of Mental Health, PLC - Personal Life Chart App, PHR-BD - Personal Health Record for Bipolar Disorder, SMS - Short Messaging Service, IAPT - Improving Access to Psychological Therapies, REDCap - Research Electronic Data Capture, CESD - Center for Epidemiologic Studies Depression, Normalised entropy - variance of time that a participant spent at a certain location, Location variance - variability in a participant’s stationary location, Number of GPS geolocation coordinate clusters - clustering technique applied to stationary states of participants, SMART – Specific Measurable Achievable Realistic Timebound, TIME – Time to Intervention for Mood Episode, bCBT - Blended Cognitive Behavioural Therapy, TAU - Treatment As Usual.** | | | | | | | | | |
| **Supplementary Table 1: Characteristics of included studies** | | | | | | | | | |

| **Study** | **Adherence to ambulatory assessment** | **Protocol Attrition** | **Ambulatory assessment/mood tracking  Procedure** | **Ambulatory assessment frequency** | **Ambulatory assessment duration** | **Active or passive** | **Analogue or digital** | **Adverse events explicitly reported** | **Acceptability/usability issues reported** |
| --- | --- | --- | --- | --- | --- | --- | --- | --- | --- |
| Anzy et al 2021 | 78.1% (SD 18.3%) | Not reported | ASERT mood self-reports weekly - 10 items that map dperessive, manic and nonspecific symptoms on a likert scale. | Weekly | 18 months | Active | Digital | No | No |
| Hidalgo-Mazzei et al 2016 | Whole sample interactions with app: 77/90 days mean (26.2). Interaction rate 1.3 times per day. | 1 month attrition: 6%, 2 month attrition: 18%, 3 month attrition: 26%. | Self-report 5 item test assessing mood, energy, sleep duration, medication adherence and irritability - daily. DSM-5 criteria for manic/depressive episodes - weekly. | Daily | 3 months | Active | Digital | No & reasons for attrition not reported | Yes |
| Hidalgo-Mazzei et al 2018 | Average daily interaction of users while using the app: 1.8 times per day (SD: 3.2). Average weekly engagement with app: 25% completed all tasks, 16% completed three quarters, 15% completed half, 44% completed <25% of tasks. | 1 month: 34.8%, 6 months: 76%, 12 months: 81% | Self-report 5 item test assessing mood, energy, sleep duration, medication adherence and irritability - daily. DSM-5 criteria for manic/depressive episodes - weekly. | Daily | 12 months | Active | Digital | No | Yes |
| Garcia-Estela et al 2022 | 22.5% never used the app, 70.9% regular users, 6.6% occasional users (engagement <12%) | 13.8% used app for >100 days, 86.2% did not.       Survival probability after 1 month 67.4% (95% CI 62.7% - 72.4%), 3 months 43% (95% CI 38.1% - 48.5%), 6 months 28% (95%CI %23.6 - %33.2) | Self-report 5 item test assessing mood, energy, sleep duration, medication adherence and irritability - daily. DSM-5 criteria for manic/depressive episodes - weekly. | Daily | 6 months | Active | Digital | No & reasons for attrition not reported | Yes |
| Bauer et al 2023 | 12% didn’t return any data. Average follow up: 227 days. | 12% didn’t return any data | ChronoRecord - daily mood via 100 point VAS, sleep, life events, menstrual data, psychiatric medication, weekly - weight. | Daily | Average follow up: 227 days | Active | Digital | No | No |
| Bos et al 2022 | 76% (number of assessments: 467, SD: 137.8, range: 197-869) | 0 | 5x EMA smartphone assessments daily - 29 items assessing monetary mood, symptoms, sleep and activities. Weekly ASRM, QIDS-SR-16 delivered via RoQua platform. | 5x daily | 4 months | Active | Digital | No | No |
| Bowden et al 2021 | 85% performed at least 1 assessment weekly | 15% | KIOS app - self report assessment of 8 different symptoms e.g sadness/pessimism and delivery of guidance in relation to symptom change. | At least one assessment weekly | 3 months | Active | Digital | No | Yes |
| Dominiak et al 2022 | 79.6% of participants had usable data. Completeness of self-report mood and sleep 27.3% and 27.5 respectively. Completeness of passive data varied from 61.1% to 100%. | 20.4% with no smartphone data over required periods | BDmon app - Passive ambulatory assessment: phone/SMS logs, participant speech information extracted from daily phone calls, Active ambulatory assessment: self report mood | Active: daily, Passive: continuous | mean: 208 days (SD: 32) | Active & Passive | Digital | No & reasons for attrition not reported | No |
| Emden et al 2021 | 33.09% still had app installed at 12 months. Median duration of install - 135 days (IQR: 111). Average rate of days on which a passive data event was sent was 73.40% (33.73%) | 66.91% | ReMAP system - Active ambulatory assessment - single mood likert scale, single item sleep scale assessing self-report sleep time, voice sample - weekly. Passive ambulatory assessment: step-count, GPS location, accelerometer - continuous. | Active: weekly, Passive: continuous | 12 months | Active & passive | Digital | No & reasons for attrition not reported | No |
| Stanislaus et al 2020 | 80% provided >1 month of passive data | 4.2% attrition in BD group | Monsenso system - Active ambulatory assessment: daily smartphone self monitoring items - mood and activity level, HDRS-17 & YMRS every 3 days. Passive ambulatory assessment: Objective smartphone data - phone usage, call/SMS logs, step count | Active: daily, Passive: continuous | Median 106 days (IQR: 48-204) | Active & passive | Active & passive | No & reasons for attrition not reported | No |
| Lee et al 2022 | 54.5% wore activity trackers for at least 30 days | Not reported | eMoodChart system - Active ambulatory assessment: self report daily mood and energy. Passive ambulatory assessment: Fitbit measuring step-count, heart rate, sleep, ambient light (android online) | Active: daily, Passive: continuous | mean: 279.7 days (SD: 263.5), median: 505 days (range: 72-1515) | Active & passive | Digital | No & reasons for attrition not reported | No |
| Born et al 2014 | Not reported | Not reported | NIMH Life Chart Methodology - prospective —twice daily mood self-rating | 2x daily | Unclear - potentially 3 years | Active | Analogue | No & reasons for attrition not reported | No |
| Lieberman et al 2011 | Not reported | Not reported | MoodChart - daily self report mood via email/online, Social Rhythm Metric - activity level over the previous 7 days | Daily | mean: 84 (range: 42-90) | Active | Digital | No & reasons for attrition not reported | No |
| Kupka et al 2005 | Not reported | Not reported | NIMH Life Chart Methodology - prospective — daily mood self-rating | Daily | 1 year | Active | Analogue | No & reasons for attrition not reported | No |
| O’Rourke et al 2021 | 196 responses per participant (range: 3-543), over an average of 145 consecutive days (range: 2-435) | Not reported | Twice daily Bipolar Disorder Symptom Scale - time/GPS stamped | 2x daily | 4+ months | Active & passive | Digital | No & reasons for attrition not reported | No |
| Tseng et al 2022 | 75% of participants performed self-assessments over 3 months. Incomplete data over 1 week: 35.87% of daily mood data, 47.45% of sleep duration data, 58.86% of GPS data. Incomplete data over 1 month: 49.50% of daily mood data, 65.38% of sleep duration data, 77.19% of GPS data. | 25% of participants did not perform self-assessments over 3 months | Active ambulatory assessment: daily mood, sleep duration. Weekly ASRM, DASS-21. Passive ambulatory assessment: GPS location | Active: daily, Passive: continuous | number of days on which participants performed self-assessments - mean: 94.25 days (median 52.5, range 2 to 398) | Active & passive | Digital | No & reasons for attrition not reported | No |
| Ebner-Priemer et al 2020 | 97% attendance at two weekly assessments, 99% for passive ambulatory assessment, 89% of daily self report mood/sleep data | 6.5% | MovisensXS system - Active ambulatory assessment: self-report mood, sleep diary.  Passive ambulatory assessment: call/text logs, GPS data, velocity, step-count. | Active: daily, Passive: continuous | 12 months | Active & passive | Digital | No & reasons for attrition not reported | No |
| Gideon et al 2016 | 37 participants made 34,830 calls over 2,436 hours. | Not reported | PRIORI system - records speech made on telephone calls. Weekly HDRS, YMRS. | Passive: continuous | 6-12 months | Active & passive | Digital | No & reasons for attrition not reported | No |
| Schneider et al 2022 | BD: 71.4% submitted data for the duration of the study, HC: 96.1% submitted data for the duration of the study | 28.6% attrition at 3 months | MINDPAX - actigraphy measuring sleep data | Passive: continuous | 3 months | Passive | Digital | No - possible adverse events highlighted via via attrition | No |
| Scharer et al 2015 | Not reported | Not reported | PLC app - daily self report mood | Daily | 18 months | Active | Digital | No & reasons for attrition not reported | No |
| van den Heuvel et al 2018 | 88.9% utilised mood chart, 66.7% utilised mood graph interface, 50% used crisis plan, messages module and medical/results module | 41% at 12 months | PHR-BD system - including 9 modules covering: medical record, medication, treatment and medical passport, general information about BD, medical results/reports, platform to send messages to appointed clinician, mood chart with daily self report mood, personal crisis plan. | Daily | 12 months | Active | Digital | No | Yes |
| Arribas et al 2018 | 81.2% | 53.1% at 12 months | AMoSS study system - Active ambulatory assessment: daily mood rating across categories of anxiety, elation, sadness, anger, irritability and energy using MoodZoom questionnaire, ASRM, QIDS-SR16, EQ-5D, GAD-7 assessed weekly. Passive ambulatory assessment: GPS, actigraphy, ambient light, call/SMS logs, heart rate via smartphone/Fitbit/GENEActive accelerometer/Proteus patch (heart rate data only for one week). | Active: daily, Passive: continuous | 3 months, with 61 participants continuing for 12 months | Active & passive | Digital | No & reasons for attrition not reported | No |
| Lewis et al 2023 | 73.5% of participants had available/sufficient data for 21 month duration analysis | 26.4% at 21 months | Bipolar Disorder Research Network using True Colours: weekly ASRM/QIDS-16-SR | Weekly | 21 months | Active | Digital | No & exact reasons for attrition not reported | No |
| McKnight et al 2017 | 92.1%. Missing data ASRM: 7.9% (IQR: 0-4.1), QIDS-SR-16: 7.8% (0-2.7) | 19.1% attrition | OXTET-1 using True Colours - ASRM/QIDS-SR-16 delivered via weekly SMS/email | Weekly | 27.5 ± 22.5 months (range: 1-81) | Active | Digital | No & exact reasons for attrition not reported | No |
| Ortiz et al 2023 | Daily e-VAS: 74.6%, Weekly PHQ-9/ASRM: 78.5%, Passive measures: 79.5% | Not reported | E-monitoring system - Active ambulatory assessment: daily rating of mood, anxiety, energy level using e-VAS. Weekly: PHQ-9, ASRM. Passive ambulatory assessment: Oura Health Oy 3d accelerometer/hyroscope measuring activity, sleep, Infrared optical pulse measuring heart rate, heart rate variability | Active: daily, Passive: continuous | 229.4 days (± 12.4) | Active & passive | Digital | No & exact reasons for attrition not reported | No |
| Aguilera et al 2017 | Percentage of texts responded to: 51.5% | Intervention median: completed 13.5 weeks of therapy prior to dropping out, Control median: completed 3 weeks of therapy prior to dropout | HealthySMS: up to 5 automated text messages including: daily mood rating, supplementation of therapy content, weekly reminders to attend CBT sessions, monthly opt out messages to end message delivery if desired. Weekly PHQ-9 for 16 weeks. | Active: daily | median: 13.5 weeks | Active | Analogue | Yes | No |
| Aikens et al 2015 | 72% completed IVR calls whenever one was attempted | 11% | Automated Interactive Voice Response telephone calls assessing symptom severity - PHQ-9 and antidepressant adherence. | Weekly | 6 months | Active | Analogue | No | No |
| Benedyk et al 2023 | Active ambulatory assessment: 74.6% (SD: 17.5), passive ambulatory assessment: 66% for depression cohort | 17.2% for depression cohort | INDICATE-N/Movisense GmbH system. Active ambulatory assessment: e-diary: twice daily fixed time smartphone-based self-ratings of mood, affect and social context via 21 e-diary items. Mood assessed via six item Multidimensional Mood Questionnaire. Participants also completed 3 intense EMA phases with 6 daily prompts for 10 days each.                           Passive ambulatory assessment: Step count, real-life GPS location tracking. | Active: daily, Passive: continuous | 6 months | Active & passive | Digital | No | No |
| Bonilla-Escribano et al 2023 | Active ambulatory assessment: 43.23% | Not reported | Memind app: 4 question daily ambulatory assessment inspired by the Salzburg Suicide Process Questionnaire. 4 questions pulled from a 32 item pool. EB2 app: GPS location, inertial sensors, physical activity, phone calls, message logs, app usage, nearby bluetooth and wifi connections. | Active: daily, Passive: continuous | 6 months | Active & passive | Digital | No | No |
| Carpenter et al 2021 | 94% | Not reported | Weekly PHQ-9 delivered via app | Weekly | 12 months | Active | Digital | No | No |
| de Angel et al 2023 | Data availability for passive RMT during the course of the study: between 20-40% | 39% | RADAR-base system PHQ-8, RSES, speech task – weekly. Heartrate, step-count, GPS location, acceleration, light levels, phone interaction, nearby Bluetooth device detection, battery level, weather, sleep, app usage metrics – continuous via Fitbit/RADAR-base app. | Active: weekly, Passive: continuous | 7 months | Active & passive | Digital | No | Yes |
| Drake et al 2013 | 68.75%: 5/16 participants did not complete any Moodscope assessments at week 12 | 20% | PHQ-9, GAD-7 - weekly. Moodscape self-rated mood - selecting which of each 20 interactive mood-adjective playing cards describes current mood. | Daily | 3 months | Active | Digital | Yes | Yes |
| Funkhouser et al 2024 | Active ambulatory assessment: 58.6% | Not reported | EARS app - Active: daily mood rating. Passive: keyboard inputs. | Active: daily, Passive: continuous | 12 months | Active & passive | Digital | No | No |
| Helmich et al 2022 | 5x daily EMA: 85%, weekly depressive symptom assessment: 98% | 30.5% | TRANS-ID Recovery System - 27 item EMA delivered 5 times a day about current feelings, activities and surroundings incorporating VAS. Weekly 14 item depressive symptom scale of SCL-90. Weekly rating of how much depressive symptoms have bothered them. | 5x daily | 4-6 months | Active | Digital | Yes | No |
| Janevic et al 2016 | 54% | Not reported | Automated Interactive Voice Response telephone calls assessing symptom severity - 6 item version of the 17 item HAM-D, PGI, WSA - and offering tailored treatment recommendations and individualised feedback. | Active: weekly | 14w | Active | Analogue - telephone | No | Yes |
| Kathan et al 2022 | Not reported | Not reported | MAIKI system - Passive ambulatory assessment: app sessions and app useage, metadata on general phone settings and phone actions, GPS data, communication information e.g contacts, call and SMS data. Active ambulatory assessment - GAD-7, PSS, PHQ-9 - weekly, items extracted from the CESD, PDD, PSQI, PHQ-2 - daily | Active: weekly, Passive: continuous | 3 months | Active & passive | Digital | No | No |
| Kline et al 2024 | 82% | 5.6% | MLife app. Active ambulatory assessment: 3 times daily modified PHQ-9 assessing symptoms over previous 4 hours, VAS of mood, optional diary entry. | Active | 3 months | Active | Digital | No | No |
| Matcham et al 2022 | >90% for active ambulatory assessment, THINC-it cognitive assessments <26%. Fitbit wear time 62.5%. 17.7% of sample had >50% data for all data types. | Active ambulatory assessment 45.4%, Passive ambulatory assessment 52.3%, Fitbit passive ambulatory assessment 32.4% at 43 weeks. | RADAR-base system PHQ-8, RSES, speech task – weekly. Heartrate, step-count, GPS location, acceleration, light levels, phone interaction, nearby Bluetooth device detection, battery level, weather, sleep, app usage metrics – continuous via Fitbit/RADAR-base app. | Active: weekly, Passive: continuous | 2 years | Active & passive | Digital | Yes | Yes |
| Mcintyre et al 2021 | 38.2% | 61.8% at 12 weeks | Mind.me system - Passive ambulatory assessment: daily call/SMS count, location variance, normalised entropy, number of GPS geolocation coordinate clusters, total distance (km), mean absolute deviation in distance (km). | Passive: continuous | 3 months | Passive | Digital | No | No |
| Meyerhoff et al 2021 | 91.2% | 8.8% | Passive Data Kit system - Passive ambulatory assessment: GPS coordinates, phone/SMS logs, duration/length, open foreground apps. | Passive: continuous | 4 months | Passive | Digital | No | Yes |
| Osgood-Hynes et al 1998 | 68% | 32% | Automated Interactive Voice Response telephone calls assessing symptom severity - 6 item version of the 17 item HAM-D, PGI, WSA - and offering tailored treatment recommendations and individualised feedback. | Weekly | 3 months | Active | Analogue | No | Yes |
| Sharp et al 2020 | PA pilot: 78.6% at week 13, BC pilot: 78.6% at week 25 | PA pilot: 21.4% at week 13, BC pilot: 21.4% at week 25 | Active Living Evday Day counselling programme - 12 weekly group educational sessions about ways to increase physical activity with Fitbit/Actigraph 3GTX+ monitoring activity levels | Passive: continuous | 3 months | Passive | Digital | No | Yes |
| Smit et al 2023 | SCL-90 weekly: median 100%, 5x daily EMA self-reports: median 87.6% | 19% at 4 months | 5x measurements per day assessing restlessness and mood for 4 months with reminder sent via text-message. SCL-90 weekly for 6 months | 5x daily | 5x daily assessments - 4 months, SCL-90 weekly for 6 months | Active | Digital | Yes | Yes |
| Vachon et al 2016 | Compliance with ambulatory assessment measures at 5 months: 81.2% (SD: 13.9) | 20% at 5 months | 2x daily self-report mood assessing affective/cognitive states relating to depression, visual analogue scale for depression, self-esteem, physical self, quality of life, coping, rumination, anxiety. | 2x daily | 5 months | Active | Digital | No | No |
| Van der Watt et al 2022 | 66% completed 16 weeks of telephone mood monitoring | 34% at 16 weeks | Weekly QIDS, ASRM administered via telephone | Weekly | 4 months | Active | Analogue | No | Yes |
| Webb et al 2022 | Not reported | 12.8% at 15 weeks | Weekly SHAPS, CES-D, BADS-SF for 15 weeks | Weekly | 15w | Active | Digital | Yes | No |
| Xia et al 2022 | GPS data mean: 77 (SD: 26), range 14-132 days. Accelerometer data mean: 74 (SD: 32), range 15-134 days. | Not reported | Beiwe system - GPS geolocation and accelerometer data | Passive: continuous | 3 months | Passive | Digital | No | No |
| Lee et al 2022 | 54.5% wore activity trackers for at least 30 days | Not reported | eMoodChart system - Active ambulatory assessment: self report daily mood and energy. Passive ambulatory assessment: Fitbit measuring step-count, heart rate, sleep, ambient light (android online) | Active: daily, Passive: continuous | mean: 279.7 days (SD: 263.5), median: 505 days (range: 72-1515) | Active & passive | Digital | No | No |
| Bilderbeck et al 2016 | Compliance/adherence to weekly measures: Both groups: 73.65%, FIMM group: 76.6% (SD 22.9%), MIMM group: 70.7% (24.6%). | Total attrition: 27% at 12 months.        Full relapse data over 12-month follow-up was available for 73% of the participants. | QIDS-SR-16, ASRM - administered weekly via TrueColours | Weekly | 12 months | Active | Digital | No - possible adverse events highlighted via via attrition | No |
| Denicoff et al 2002 | Not reported. | Total attrition over 3 treatment phases: 44% attrition at 3 years | NIMH Life Chart Methodology - prospective — daily mood self-rating | Daily | 3 years | Active | Analogue | No | No |
| Faurholt-Jepsen et al 2015 | >93% of patients randomised to intervention group self reported on a daily basis | Intervention group: 3% attrition over 6 months, control group: 3% attrition over 6 months. | Daily smartphone self monitoring - mood, sleep duration, medication taken, activity, irritability, mixed mood, cognitive problems, alcohol consumption, stress, menstruation, individualised EWS | Daily | 6 months | Active & Passive | Digital | No - possible adverse events highlighted via via attrition | No |
| Faurholt-Jepsen et al 2019 | Over 9 months patients in the intervention group adhered to the daily self-monitoring 72.6% of the days. | Intervention group: 7% attrition at 9 months, control group: 7% attrition at 9 months. | Daily smartphone self monitoring items - mood, sleep duration, medication taken, activity, irritability, mixed mood, cognitive problems, alcohol consumption, stress, menstruation, individualised EWS, anxiety, self-defined personal parameters, free-text note.                 Objective smartphone data - phone usage, social activity, step count, GPS location | Active: daily, Passive: continuous | 9 months | Active & Passive | Digital | No - possible adverse events highlighted via via attrition | No |
| Faurholt-Jepsen et al 2020 | 80.6% adherence to daily self-monitoring in intervention group over 6 months. | Total attrition: 35% at 6 months, Intervention group: 22%, Control group: 53%. | Daily smartphone self monitoring items - mood, sleep duration, medication taken, activity, irritability, mixed mood, cognitive problems, alcohol consumption, stress, menstruation, individualised EWS, anxiety, self-defined personal parameters, free-text note. Objective smartphone data - phone usage, social activity, step count, GPS location | Active: daily, Passive: continuous | 6 months | Active & Passive | Digital | No - possible adverse events highlighted via via attrition | No |
| Gliddon et al. 2018 | Control group: 89% accessed discussion forum, MoodSwings group: 86% accessed the modules, MoodSwings-Plus: 74% accessed the tools. | Total attrition: 9% at 12 months, Control group: 6%, MoodSwings group: 7%, MoodSwings-Plus: 13% | Online mood-monitoring via MoodSwings & MoodSwings-Plus websites | Dependent on individual participant’s use of application | 12 months | Active | Digital | No - possible adverse events highlighted via via attrition | No |
| Lauder et al 2015 | 48% completed all 5 MoodSwings modules, 86.2% completed at least 2 modules, 75.4% completed at least 3 modules | Total attrition: 92% at 12 months, Intervention group: 94%, Control group: 91% | Online mood-monitoring via MoodSwings & MoodSwings-Plus | Dependent on individual participant’s use of application | 12 months | Active | Digital | No - possible adverse events highlighted via via attrition | No |
| Castle et al 2010 | Not reported | Total attrition: 14% at 12 months, Intervention group: 24%, Control group: 5% | Weekly telephone calls – weekly for 12 weeks. | Weekly | 12 weeks | Active | Analogue | No - possible adverse events highlighted via via attrition | No |
| Goulding et al 2022 | The mean (SE) percentage of daily check-ins completed during weeks 1 through 4 was 78% (3%), 74% (3%), 71% (3%), and 64% (3%), respectively, 66% (3%) during week 6, and 47% (4%) during week 16. | Intervention group: 15% attrition at 4 months, Control group: 15% attrition at 4 months. | Smartphone based self management intervention - daily and weekly check-ins for weeks 1-16. Daily - adherence, sleep, duration, routine, wellness levels. Weekly - symptom severity scoring for all individual DSM-IV mood symptoms. | Daily | 4 months | Active | Digital | No - possible adverse events highlighted via via attrition | No |
| Petzold et al. 2019 | Not reported | Total: 54.8% over 2 years, Intervention group: 51% attrition over 2 years, Control group: 59% attrition over 2 years. | ChronoRecord - daily mood, sleep, life events, menstrual data, psychiatric medication, weekly - weight. | Daily | 12.5 months | Active | Digital | No - possible adverse events highlighted via via attrition | No |
| Van den Berg et al 2023 | Not reported | Total attrition: 18% at 4 months, Intervention group: 16% at 4 months, Control group: 20% at 4 months | NIMH Life Chart Methodology - daily mood and anxiety self-rating. | Daily | 4 months | Active | Digital | No & reasons for attrition not reported | No |
| Goldberg et al. 2006 | Not reported | ~50% attrition at 3 months, >90% attrition at 6.5 months | NIMH Life Chart Methodology - daily mood self-rating | Daily | 6.5 months | Active | Analogue | No & reasons for attrition not reported | No |
| Langosch et al 2008 | Not reported | 55% attrition at 12 months | NIMH Life Chart Methodology - daily mood self-rating | Daily | 12 months | Active | Analogue | Yes | No |
| Leverich et al 2006 | Not reported | Not reported | NIMH Life Chart Methodology - daily mood self-rating | Daily | 12 months | Active | Analogue | No | No |
| Lieberman et al 2010 | Online group rated 44.3 days, standard group rated 20.4 days. Online group entered complete data on 55.2% of days compared to the standard group of 27.7% of days. | Total attrition: 46% attrition at 3 months, paper chart: 68% attrition at 3 months, online chart: 22% attrition at 3 months. | NIMH Life Chart Methodology - prospective — daily mood self-rating / online Life Chart adaptation | Daily | 3 months | Active | Analogue vs Digital | No | No |
| Depp et al 2012 | Mean observations in paper and pen charting 51.2 (sd: 27.1), mean observations in smartphone charting 72.3 (sd = 61.5). | 34% attrition at 3 months | NIMH Life Chart Methodology - prospective — daily mood self-rating via paper and pen and via smartphone | Daily | 3 months | Active | Analogue vs Digital | No & reasons for attrition not reported | No |
| Pahwa et al 2023 | KIOS: 84.4%, eMoods: 54% | KIOS: 12.30%, eMoods: 26.31% at 52 weeks | KIOS app - self report assessment of 8 different symptoms e.g sadness/pessimism and delivery of guidance in relation to symptom change.    eMoods app – self report mood and symptoms diary tracking daily outlook, motivation, habits, sleep, medications etc | Daily | 12 months | Active | Digital | No & reasons for attrition not reported | Yes |
| Aikens et al 2022 | 22% in intervention arm completed <50% of scheduled calls | Total attrition: 14%, intervention 17%, control 10% | Automated Interactive Voice Response telephone calls assessing symptom severity - PHQ-9 and antidepressant adherence. | Weekly | 12 months | Active | Analogue | Yes | No |
| van Genugten et al. 2021 | 32% did not receive treatment or did not provide any weekly ambulatory assessment reports | Intervention attrition: 32% | Daily self-monitoring of mood state via VAS, cognitions, activities, social interaction, and sleep via a smartphone app. Ambulatory assessment protocol varied over course of the study, one some days 3x daily mood ratings. | Daily | 12w | Active | Digital | No | No |
| Arean et al 2016 | 65.2% used the survey app, 56.6% provided at least one follow up assessment. | 70% | Active ambulatory assessment: PHQ-9, SDS, Global Impression of Change Scale, 3 question sleep assessment, Mental Health Services Used - weekly. PHQ-2 - daily. Passive ambulatory assessment - time of call/SMS, call duration, SMS length, activity type, distance travelled. | Active: daily, Passive: continuous | 12w | Active & passive | Digital | No | No |
| Pratap et al 2018 | 18.7% downloaded treatment app, 74/389 of hispanic/latino subset. Full results not reported. | 74% | Active ambulatory assessment: PHQ-9, SDS , Global Impression of Change Scale, 3 question sleep assessment, Mental Health Services Used - weekly. PHQ-2 - daily. Passive ambulatory assessment - time of call/SMS, call duration, SMS length, activity type, distance travelled. | Active: daily, Passive: continuous | 12w | Active & passive | Digital | No | No |
|  |  |  |  |  |  |  |  |  |  |
| Dai et al 2022 | 83.9% | 17.1% | Wearable activity tracker (Fitbit) measuring sedentary minutes, lightly active minutes, minutes of heart rate zone in fat-burn, minutes of heart rate zone in cardio, total walking distance, activity calories, minutes awake in main sleep, restless count in main sleep, efficiency in main sleep, time in bed of main sleep. | Passive: continuous | 6 months | Passive | Digital | No | No |
| Tonning et al 2021 | 82.7% in intervention arm | Total attrition: 17.5%, intervention: 20%, control: 15% | Monsenso system plus: 1. study nurse reviewing data and contacting patients if sign of deterioration to offer advice 2. self-monitored data graphically visualised 3. smartphone based CBT modules | Active: daily, Passive: continuous | 6 months | Active & passive | Digital | Yes | No |
| Frank et al 2022 | Passive ambulatory assessment mean: 93.32% across 16 weeks | Total attrition: 7.4% | Active ambulatory assessment: Daily VAS rating of mood. Passive ambulatory assessment: behaviour monitoring | Passive: continuous | 16w | Active & passive | Digital | Yes | No |
| Hunkeler et al 2012 | ~87% entered any monitoring data over the first 6 months, ~45% entered any monitoring data over the second 6 months | Total attrition: 16%, intervention attrition: 22%, control attrition: 12% | Personalised self-monitoring via eCare for Moods - tracking health-related disability, medication adherence, side effects, alcohol and drug use, new symptoms, early warning signs. Graphs of monitoring data displayed over time. | Weekly | 24 months | Active | Digital | No | No |
| Klein et al 2016 | N/A | Total attrition: 16%, Intervention attrition: 16%, control attrition: 15% | Weekly contact by trained email supporter - short feedback based on participants’ program usage over the past week. Feedback is relatively generic in nature and does not apply therapeutic strategies in detail. PHQ-9 biweekly. | 2x weekly | 12w | Passive | Digital | Yes | No |
| Tuvey et al 2023 | 86% responded to at least one text by week 6. | 15% at 6 weeks | Intervention arm 1: Annie Secure Messaging (SM) system – promoting antidepressant adherence with texts around efficiacy and AEs, Intervention arm 2: Annie SM system with coaching – weekly coaching call with a study member to review weekly data reported via Annie looking at trends over time – participants asked to reflect on trends and identify any self-management issues | Active: daily | 12w | Active | Digital - text message | No | No |
| White et al 2023 | Intervention adherence: 67%, control adherence: 80% | Total attrition: 3%, Intervention attrition: 4%, control attrition: 2% | PHQ-8, RSES, speech task – weekly. Heartrate, step-count, GPS location, app usage metrics – continuous via Fitbit/RADAR-base app | Active: weekly, Passive: continuous | 12w | Passive | Digital | Yes | Yes |
| **ASRM - Altman Self Rating Mania Scale, QIDS - Quick Inventory of Depressive Symptomatology, IDS - Inventory of Depressive Symptomatology, HAM-D - Hamilton Depression Rating Scale, CGI-BP - Clinical Global Impressions - Bipolar Scale, YMRS - Young Mania Rating Scale, SCID - Structured Clinical Interview for DSM-IV, PSS - Perceived Stress Scale, FAST - Functional Assessment Short Test, WHOQoL-BREF - World Health Organisation Quality of Life Scale, BDI - Beck Depression Inventory, HDRS6 - 6 item Hamilton Depression Rating Scale, MARS - Medication Adherence Rating Scale, RRS - Ruminitive Response Scale, PSWQ - Penn State Worry Questionnaire, BAS - Behavioural Activation Scale, VSS-A - Verona Satisfaction Scale-Affective Disorder, BAI - Beck Anxiety Inventory, ALS-18 - Affect Lability Score Short Version, Life-Rift - Level of general functioning and coping: Longitudinal Interval Follow-up Evaluation, BHS - Beck Hopelessness Scale, GSE - General Self-Efficacy Scale, HLOC - Health Locus of Control Scale, PICS - Perceived Involvement in Care Scales, RBANS - Repeatable Battery for the Assessment of Neuropsychological Status, NOS - Not Otherwise Specified, NIMH - National Institute of Mental Health, PHQ-9 - Patient Health Questionnaire-9, PHQ-2 - Patient Health Questionnaire - 2, VAS - Visual Analog Scale, SDS - Sheehan Disability Scale, EMA - Ecological Momentary Assessment, BD - Bipolar Disorder, UD - Unipolar Depression, SMS - Short Messaging Service, CBT - Cognitive Behavioral Therapy** | | | | | | | | | |
| **Supplementary Table 2: Ambulatory assessment protocols of included studies** | | | | | | | | | |

**Supplementary Table 3: Risk of bias assessments for included randomised and non-randomised studies**

| **Risk of bias assessments for included non-randomised studies** | | | | | | | | |
| --- | --- | --- | --- | --- | --- | --- | --- | --- |
| **Study** | **Risk of bias criteria** | | | | | | | |
|  | **Confounding bias** | **Selection of participants into the study** | **Classification of interventions** | **Deviation from intended intervention** | **Missing data** | **Measurement of outcomes** | **Selection of reported result** | **Total no of low risk domains** |
| Anzy et al 2021 | Low risk | Unclear | Low risk | Unclear | Unclear | Unclear | Low risk | 3 |
| Hidalgo-Mazzei et al 2016 | Low risk | Low risk | Low risk | Low risk | Low risk | High risk | Low risk | 6 |
| Hidalgo-Mazzei et al 2018 | Low risk | Low risk | Low risk | High risk | High risk | High risk | Low risk | 4 |
| Garcia-Estela et al 2022 | Low risk | Low risk | Low risk | High risk | High risk | High risk | Low risk | 4 |
| Bauer et al 2023 | Low risk | Unclear | Low risk | Low risk | Low risk | High risk | Low risk | 5 |
| Bos et al 2022 | Low risk | Low risk | Low risk | Low risk | Low risk | High risk | Low risk | 6 |
| Bowden et al 2021 | Low risk | Unclear | Low risk | Low risk | Low risk | High risk | Low risk | 5 |
| Dominiak et al 2022 | Low risk | Low risk | Low risk | Low risk | Low risk | High risk | Low risk | 6 |
| Emden et al 2021 | Low risk | Low risk | Low risk | High risk | High risk | High risk | Low risk | 4 |
| Stanislaus et al 2020 | Low risk | Unclear | Low risk | Low risk | Low risk | Low risk | Low risk | 6 |
| Lee et al 2022 | Low risk | Unclear | Low risk | High risk | High risk | High risk | Low risk | 5 |
| Born et al 2014 | Low risk | High risk | Low risk | Unclear | Unclear | High risk | Low risk | 5 |
| Lieberman et al 2011 | High risk | High risk | Low risk | Unclear | Unclear | High risk | Low risk | 2 |
| Kupka et al 2005 | Low risk | Unclear | Low risk | Unclear | Unclear | High risk | Low risk | 3 |
| O’Rourke et al 2021 | Low risk | High risk | Low risk | High risk | Unclear | High risk | Low risk | 3 |
| Tseng et al 2022 | Low risk | Unclear | Low risk | High risk | High risk | High risk | Low risk | 5 |
| Ebner-Priemer et al 2020 | Low risk | Low risk | Low risk | Low risk | Low risk | High risk | Low risk | 6 |
| Gideon et al 2016 | Low risk | Unclear | Low risk | Unclear | Unclear | High risk | Low risk | 3 |
| Schneider et al 2022 | Low risk | Unclear | Low risk | Low risk | Low risk | High risk | Low risk | 6 |
| Scharer et al 2015 | Low risk | Unclear | Low risk | Unclear | Unclear | High risk | Low risk | 3 |
| van den Heuvel et al 2018 | Low risk | Low risk | Low risk | High risk | High risk | High risk | Low risk | 4 |
| Arribas et al 2018 | Low risk | Unclear | Low risk | High risk | High risk | High risk | Low risk | 3 |
| Lewis et al 2023 | Low risk | Unclear | Low risk | Low risk | Low risk | Low risk | Low risk | 6 |
| McKnight et al 2017 | Low risk | Unclear | Low risk | Low risk | Low risk | High risk | Low risk | 5 |
| Ortiz et al 2023 | Low risk | Unclear | Low risk | Unclear | Unclear | High risk | Low risk | 3 |
| Aguilera et al 2017 | Low risk | Unclear | Low risk | Unclear | High risk | High risk | Low risk | 3 |
| Aikens et al 2015 | Low risk | Low risk | Low risk | Low risk | Low risk | High risk | Low risk | 6 |
| Benedyk et al 2023 | Low risk | Unclear | Low risk | High risk | High risk | High risk | Low risk | 3 |
| Bonilla-Escribano et al 2023 | Low risk | Low risk | High risk | High risk | High risk | High risk | Low risk | 3 |
| Carpenter et al 2021 | Unclear | Unclear | Low risk | Low risk | High risk | Unclear | Low risk | 3 |
| de Angel et al 2023 | Low risk | Low risk | Low risk | High risk | High risk | High risk | Low risk | 4 |
| Drake et al 2013 | Low risk | Low risk | Low risk | Low risk | Low risk | High risk | Low risk | 6 |
| Emden et al 2021 | Low risk | High risk | High risk | High risk | High risk | High risk | Low risk | 2 |
| Funkhouser et al 2024 | Low risk | High risk | Low risk | Low risk | High risk | High risk | High risk | 3 |
| Helmich et al 2022 | Low risk | High risk | Low risk | Low risk | High risk | High risk | Low risk | 4 |
| Janevic et al 2016 | Low risk | Low risk | Low risk | High risk | High risk | High risk | High risk | 3 |
| Kathan et al 2022 | Low risk | Unclear | Low risk | Unclear | Unclear | High risk | Low risk | 3 |
| Kline et al 2024 | Low risk | High risk | Low risk | Low risk | High risk | High risk | High risk | 3 |
| Lee et al 2022 | Low risk | Unclear | Low risk | High risk | High risk | High risk | Low risk | 5 |
| Matcham et al 2022 | Low risk | Low risk | Low risk | High risk | High risk | High risk | Low risk | 4 |
| Mcintyre et al 2021 | Low risk | Unclear | Low risk | High risk | High risk | High risk | Low risk | 3 |
| Meyerhoff et al 2021 | Low risk | High risk | Low risk | Low risk | Low risk | High risk | Low risk | 5 |
| Osgood-Hynes et al 1998 | Low risk | Low risk | Low risk | High | High risk | High risk | Low risk | 4 |
| Sharp et al 2020 | Low risk | Unclear | Low risk | High risk | High risk | High risk | Low risk | 3 |
| Smit et al 2023 | Low risk | High risk | Low risk | High risk | High risk | High risk | Low risk | 3 |
| Vachon et al 2016 | Low risk | Unclear | Low risk | Low risk | Low risk | High risk | Low risk | 5 |
| Van der Watt et al 2022 | Low risk | Unclear | Low risk | High risk | High risk | High risk | Low risk | 3 |
| Webb et al 2022 | Low risk | Unclear | Low risk | Low risk | Low risk | High risk | Low risk | 5 |
| Xia et al 2022 | Low risk | Unclear | Low risk | High risk | Unclear | High risk | High risk | 2 |
| **Risk of bias assessments for included randomised studies** | | | | | | | | |
| **Study** | **Risk of bias criteria** | | | | | | | |
|  | **Random sequence generation** | **Allocation concealment** | **Blinding of participants and personnel** | **Blinding of outcome assessment** | **Incomplete outcome data** | **Selective reporting** | **Other sources of bias** | **Total no of low risk domains** |
| Bilderbeck et al 2016 | Low risk | Low risk | High risk | High risk | Low risk | Low risk | Low risk | 5 |
| Denicoff et al 2002 | Unclear | High risk | High risk | Low risk | Low risk | Low risk | Low risk | 4 |
| Faurholt-Jepsen et al 2015 | Low risk | Low risk | High risk | Low risk | Low risk | Low risk | Low risk | 6 |
| Faurholt-Jepsen et al 2019 | Low risk | Low risk | High risk | Low risk | Low risk | Low risk | Low risk | 6 |
| Faurholt-Jepsen et al 2020 | Low risk | Low risk | High risk | Low risk | Low risk | Low risk | Low risk | 6 |
| Lauder et al. 2015 | Low risk | Low risk | Low risk | Low risk | High risk | Low risk | Low risk | 6 |
| Gliddon et al. 2018 | Low risk | Low risk | High risk | Low risk | Low risk | Low risk | Low risk | 6 |
| Castle et al. 2010 | Low risk | Unclear | High risk | Unclear | Low risk | Low risk | Low risk | 4 |
| Goulding et al 2022 | Low risk | Low risk | High risk | Low risk | Low risk | Low risk | Low risk | 6 |
| Petzold et al. 2019 | Low risk | Low risk | High risk | Low risk | Low risk | Low risk | Low risk | 6 |
| Van den Berg et al 2023 | Unclear | Unclear | High risk | Low risk | Low risk | Low risk | Low risk | 4 |
| Goldberg et al. 2006 | Unclear | Unclear | Low risk | Low risk | Unclear | Low risk | Low risk | 4 |
| Langosch et al 2008 | Unclear | High risk | High risk | High risk | Low risk | Low risk | Low risk | 3 |
| Leverich et al 2006 | Unclear | Low risk | Unclear | Low risk | High risk | High risk | Low risk | 3 |
| Lieberman et al 2010 | Unclear | Unclear | Unclear | High risk | Low risk | Low risk | Low risk | 3 |
| Depp et al 2012 | Unclear | Low risk | Low risk | Low risk | Low risk | Low risk | Low risk | 6 |
| Pahwa et al 2023 | Low risk | High risk | High risk | High risk | Low risk | Low risk | Low risk | 4 |
| Aikens et al 2022 | Low risk | High risk | High risk | High risk | Low risk | Low risk | Low risk | 4 |
| van Genugten et al. 2021 | Low risk | Low risk | High risk | High risk | Low risk | Low risk | Low risk | 5 |
| Arean et al 2016 | High risk | High risk | High risk | High risk | Low risk | Low risk | Low risk | 3 |
| Pratap et al 2018 | High risk | High risk | High risk | High risk | Low risk | Low risk | Low risk | 3 |
| Dai et al 2022 | Low risk | High risk | High risk | Low risk | Low risk | Low risk | Low risk | 5 |
| Tonning et al 2021 | Low risk | Low risk | High risk | Low risk | Low risk | Low risk | Low risk | 6 |
| Frank et al 2022 | Unclear | Unclear | High risk | High risk | Low risk | Low risk | Low risk | 3 |
| Hunkeler et al 2012 | Low risk | Low risk | High risk | High risk | Low risk | Low risk | Low risk | 5 |
| Klein et al 2016 | Low risk | Low risk | High risk | Low risk | Low risk | Low risk | Low risk | 6 |
| Tuvey et al 2023 | Unclear | Unclear | High risk | Unclear | Low risk | Low risk | Low risk | 3 |
| White et al 2023 | Low risk | Low risk | High risk | High risk | Low risk | Low risk | Low risk | 5 |
| **Supplementary Table 3: Risk of bias assessments for included randomised and non-randomised studies** | | | | | | | | |
| **Supplementary Table 4: Adverse events of included studies** | | | | | | | | |

| **Adverse event type** | **Nature of event** | **Relatedness to ambulatory assessment** | **Number of studies reported in** | **Study reported in** | **Affected n** | **Total n** | **Percentage** |
| --- | --- | --- | --- | --- | --- | --- | --- |
| Negative psychological symptoms - too burdensome/stressful | Moderate | Probably related | 9 | van den Heuvel et al 2018 | 9 | 66 | 0.14 |
|  |  |  |  | Drake et al 2013 | 1 | 20 | 0.05 |
|  |  |  |  | Helmich et al 2022 | 12 | 41 | 0.29 |
|  |  |  |  | Matcham et al 2022 | 10 | 623 | 0.02 |
|  |  |  |  | Bilderbeck et al 2016 | 1 | 121 | 0.01 |
|  |  |  |  | Faurholt-Jepsen et al 2019 | 6 | 97 | 0.06 |
|  |  |  |  | Goulding et al 2022 | 13 | 124 | 0.10 |
|  |  |  |  | Petzold et al 2019 | 1 | 39 | 0.03 |
|  |  |  |  | Tonning et al 2021 | 7 | 120 | 0.06 |
| Negative psychological symptoms - subjective worsening of mood | Moderate/severe | Probably related | 6 | van den Heuvel et al 2018 | 1 | 66 | 0.02 |
|  |  |  |  | Drake et al 2013 | 2 | 20 | 0.10 |
|  |  |  |  | Helmich et al 2022 | 9 | 59 | 0.15 |
|  |  |  |  | Matcham et al 2022 | 9 | 623 | 0.01 |
|  |  |  |  | Smit et al 2023 | 33 | 56 | 0.59 |
|  |  |  |  | Osgood-Hynes et al 1998 | 1 | 41 | 0.02 |
| Suicidal ideation | Severe | Possibly related | 2 | Gliddon et al 2018 | 1 | 304 | 0.00 |
|  |  |  |  | Bonilla-Escribano et al 2023 | 23 | 374 | 0.06 |
| Self harm | Severe | Possibly related | 4 | Langosch et al 2008 | 1 | 38 | 0.03 |
|  |  |  |  | Webb et al 2022 | 1 | 39 | 0.03 |
|  |  |  |  | Gliddon et al 2018 | 2 | 304 | 0.01 |
|  |  |  |  | Bonilla-Escribano et al 2023 | 45 | 374 | 0.12 |
| Hospitalisation | Severe | Possibly related | 3 | Bilderbeck et al 2016 | 12 | 121 | 0.10 |
|  |  |  |  | van den Heuvel et al 2018 | 4 | 66 | 0.06 |
|  |  |  |  | Bonilla-Escribano et al 2023 | 19 | 374 | 0.05 |
| Relapse | Severe | Possibly related | 3 | Bilderbeck et al 2016 | 50 | 121 | 0.41 |
|  |  |  |  | Petzold et al 2019 | 23 | 39 | 0.59 |
|  |  |  |  | Smit et al 2023 | 19 | 37 | 0.51 |
| Seizure | Severe | Not related | 1 | Webb et al 2022 | 1 | 39 | 0.03 |
| Pregnancy | Severe | Not related | 1 | Gliddon et al 2018 | 2 | 304 | 0.01 |
| Orthostatic dysregulation | Moderate | Not related | 1 | Langosch et al 2008 | 16 | 38 | 0.42 |
| Sedation | Moderate | Not related | 1 | Langosch et al 2008 | 12 | 38 | 0.32 |
| Dry mouth | Mild | Not related | 1 | Langosch et al 2008 | 8 | 38 | 0.21 |
| Headache | Moderate | Not related | 1 | Langosch et al 2008 | 8 | 38 | 0.21 |
| Palpitations | Moderate | Not related | 1 | Langosch et al 2008 | 5 | 38 | 0.13 |
| Accommodation disturbances | Moderate | Not related | 1 | Langosch et al 2008 | 4 | 38 | 0.11 |
| Weight gain | Moderate | Not related | 1 | Langosch et al 2008 | 4 | 38 | 0.11 |
| Vomiting | Moderate | Not related | 1 | Langosch et al 2008 | 3 | 38 | 0.08 |
| Obstipation | Moderate | Not related | 1 | Langosch et al 2008 | 3 | 38 | 0.08 |
| Paraesthesia | Moderate | Not related | 1 | Langosch et al 2008 | 4 | 38 | 0.11 |
| Flu | Moderate | Not related | 1 | Langosch et al 2008 | 4 | 38 | 0.11 |
| Oedema | Moderate | Not related | 1 | Langosch et al 2008 | 4 | 38 | 0.11 |
| Restlessness | Moderate | Not related | 1 | Langosch et al 2008 | 3 | 38 | 0.08 |
| Stomach spasm | Moderate | Not related | 1 | Langosch et al 2008 | 3 | 38 | 0.08 |
| Urogenital Infection | Moderate/severe | Not related | 1 | Langosch et al 2008 | 2 | 38 | 0.05 |
| Atopia | Modeate | Not related | 1 | Langosch et al 2008 | 2 | 38 | 0.05 |
| Death | Severe | Not related | 1 | Matcham et al 2022 | 4 | 623 | 0.01 |
| **Supplementary Table 4: Adverse events of included studies** | | | | | | | |

Supplementary Information 2: Systematic review search strategy

Search performed 3/3/23. The search strategy was trialled on one database first and then refined subsequently. The search results were uploaded to Rayyan (81). Search terms were determined based on discussion between researchers, previous reviews and consultation with specialist librarians. The search was performed from inception to 3/3/23. The search was updated on 28/10/24.

**Number of abstracts original search 3/3/23:**

Medline: 2984

Embase: 4827

PsychINFO: 3346

SCOPUS: 2321

IEE Xplore: 615

Proquest dissertations and theses global: 2697

Proquest SciTech Collection: 3489

Total: 20,279

Full text review: 565

**Number of abstracts of updated search 3/3/24 – 28/10/24:**

Medline: 494

Embase: 920

PsychINFO: 364

SCOPUS: 1088

IEE Xplore: 99

Proquest dissertations and theses global: 0

Proquest SciTech Collection: 469

Total prior to deduplication: 3236

Auto-deduplicated: 1119

Total: 2117

Full text review: 193

**Published literature:**

| **OVID Medline** | |
| --- | --- |
| 1 | exp bipolar disorder/ OR exp depression OR exp mania/ |
| 2 | (((bipolar or bi polar) adj5 (disorder$ or depress$)) or ((cyclothymi$ or rapid or ultradian) adj5 cycl$) or hypomani$ or mania$ or manic$ or mixed episode$ or rcbd).mp |
| 3 | ('Depressive Disorder' OR 'Depression' OR 'dysthymi*' OR 'affective disorder' OR 'affective disorders' OR 'mood disorder' OR 'mood disorders' OR 'depression*' OR 'depressive*' OR 'dysthymic disorder').mp |
| 4 | 1 OR 2 OR 3 |
| 5 | ('self monitor*' or 'self assess*' or 'self manag*' or 'self record*' or 'self surveillance' or 'patient* monitor*' or 'measurement technolog*' or 'telemonitor*' or 'remote monitor*' or 'passive monitor*' or 'active monitor*' or 'mood track*' or 'mood monitor*' or 'experience sampl*' or 'ecological momentary assessment').mp |
| 6 | 4 adj10 5 |

https://ovidsp.ovid.com/ovidweb.cgi?T=JS&NEWS=N&PAGE=main&SHAREDSEARCHID=10Q4IjupCc3HoHvVXInK959r2tcDy9vTlJlQsifUVfSVSyNrczwzGqVCqql3svtpo

| **OVID EMBASE** | |
| --- | --- |
| 1 | bipolar disorder/ or bipolar depression/ or bipolar I disorder/ |
| 2 | depression assessment/ or treatment resistant depression/ or minor depression/ or chronic depression/ or postnatal depression/ or atypical depression/ or antenatal depression/ or adolescent depression/ or "mixed mania and depression"/ or post-stroke depression/ or endogenous depression/ or major depression/ or recurrent brief depression/ or depression/ or bipolar depression/ or perinatal depression/ or agitated depression/ or organic depression/ |
| 3 | "mixed mania and depression"/ or mania/ or bipolar mania/ |
| 4 | (((bipolar or bi polar) adj5 (disorder$ or depress$)) or ((cyclothymi$ or rapid or ultradian) adj5 cycl$) or hypomani$ or mania$ or manic$ or mixed episode$ or rcbd).mp. |
| 5 | ('Depressive Disorder' or 'Depression' or 'dysthymi*' or 'affective disorder' or 'affective disorders' or 'mood disorder' or 'mood disorders' or 'depression*' or 'depressive*' or 'dysthymic disorder').mp. |
| 6 | 1 OR 2 OR 3 OR 4 OR 5 |
| 7 | ('self monitor*' or 'self assess*' or 'self manag*' or 'self record*' or 'self surveillance' or 'patient* monitor*' or 'measurement technolog*' or 'telemonitor*' or 'remote monitor*' or 'passive monitor*' or 'active monitor*' or 'mood track*' or 'mood monitor*' or 'experience sampl*' or 'ecological momentary assessment').mp. |
| 8 | 6 adj10 7 |

https://ovidsp.ovid.com/ovidweb.cgi?T=JS&NEWS=N&PAGE=main&SHAREDSEARCHID=2Ofrc9VijRp6L40USOJFeEn3I1bHOmCW8O1Hzfz13xklneo3jW1767QyyDQMHnVDj

| **OVID PsychINFO** | |
| --- | --- |
| 1 | Bipolar Disorder/ or Bipolar II Disorder/ or Bipolar I Disorder/ or Mania/ |
| 2 | Major Depression/ or Endogenous Depression/ or Postpartum Depression/ or Recurrent Depression/ or "Depression (Emotion)"/ or Reactive Depression/ or Late Life Depression/ or Atypical Depression/ or Treatment Resistant Depression/ or "Long-term Depression (Neuronal)".mp. |
| 3 | (((bipolar or bi polar) adj5 (disorder$ or depress$)) or ((cyclothymi$ or rapid or ultradian) adj5 cycl$) or hypomani$ or mania$ or manic$ or mixed episode$ or rcbd).mp |
| 4 | 'Depressive Disorder' OR 'Depression' OR 'dysthymi*' OR 'affective disorder' OR 'affective disorders' OR 'mood disorder' OR 'mood disorders' OR 'depression*' OR 'depressive*' OR 'dysthymic disorder' |
| 5 | 1 OR 2 OR 3 OR 4 |
| 6 | ('self monitor*' or 'self assess*' or 'self manag*' or 'self record*' or 'self surveillance' or 'patient* monitor*' or 'measurement technolog*' or 'telemonitor*' or 'remote monitor*' or 'passive monitor*' or 'active monitor*' or 'mood track*' or 'mood monitor*' or 'experience sampl*' or 'ecological momentary assessment') |
| 7 | 5 adj10 6 |

https://ovidsp.ovid.com/ovidweb.cgi?T=JS&NEWS=N&PAGE=main&SHAREDSEARCHID=7WPhKe8RR9Athylx2jCCPdAkgbQlgcrdVpxl6NGPhskh73E8wr3X16vfACAP9Q54Y

**SCOPUS:**

TITLE-ABS-KEY({self monitor*} OR {self-monitor*} OR {self-assess*} OR {self manag*} OR {self-manag*} OR {self record*} OR {self-record*} OR {self surveillance} OR {self-surveillance} OR {patient* monitor*} OR {patient*-monitor*} OR {measurement technolog*} OR {measurement-technolog*} OR {telemonitor*} OR {remote monitor*} OR {remote-monitor*} OR {passive monitor*} OR {passive-monitor*} OR {active monitor*} OR {active-monitor*} OR {mood track*} OR {mood-track*} OR {mood monitor*} OR {mood-monitor*} OR {experience sampl*} OR {ecological momentary assessment}) W/10 ({Bipolar disorder*} OR {Manic depress*} OR {Manic-depress*} OR {Bipolar affective psychos*} OR {Bipolar depress*} OR {Manic disorder*} OR (82) OR {depressive disorder*} OR {major depressive disorder*} OR (82) OR {affective disorder*} OR {mood disorder*})

**IEE XPLORE:**

('self monitor' OR 'self monitoring' OR 'self assess' OR 'self assessment' OR 'self manage' OR 'self management' OR 'self record' OR 'self recording' OR 'self surveillance' OR 'patient monitor' OR 'patient monitoring' OR 'measurement technology' OR 'telemonitor' OR 'telemonitoring' OR 'remote monitor' OR 'remote monitoring' OR 'passive monitor' OR 'passive monitoring' OR 'active monitor*' OR 'mood track*' OR 'mood monitor*' OR 'experience sample' OR 'experience sampling' OR 'ecological momentary assessment') NEAR/10 ('Bipolar*' OR 'Manic disorder*' OR 'depressi*' OR 'affective disorder*' OR 'mood disorder*')

**Google scholar search:** An additional search of the first 15 pages of Google Scholar was conducted (search terms: ‘mood track’, ‘ecological momentary assessment’, ‘monitoring’, ‘remote monitoring’, ‘active monitor’, ‘passive monitor’, ‘experience sample’, ‘experience sampling’)

Finally, subject experts were approached to identify additional articles.

**Grey Literature:**

**ProQuest Dissertations & Theses Global:**

(("self monitor" OR "self monitoring" OR "self monitors") OR ("self assess" OR "self assessed" OR "self assessment") OR ("self manage" OR "self managed" OR "self managing") OR ("self record" OR "self recorded" OR "self recording") OR “self surveillance” OR “patient* monitor*” OR ("measurement technologies" OR "measurement technology") OR “telemonitor*” OR ("remote monitoring") OR ("passive monitoring") OR ("active monitoring") OR “mood track*” OR “mood monitor*” OR ("experience sampling") OR “ecological momentary assessment”) NEAR/10 (("bipolar disorder" OR "bipolar disorders") OR ("manic depression" OR "manic depressive") OR “Bipolar affective psychos*” OR ("bipolar depression") OR “Manic disorder*” OR “depressi*” OR ("depressive disorder") OR “major depressive disorder*” OR “depression” OR ("affective disorder" OR "affective disorders") OR ("mood disorder" OR "mood disorders"))

http://abc.cardiff.ac.uk/login?url=https://www.proquest.com/search/2332884?accountid=9883

https://www.proquest.com/pqdtglobal

**ProQuest SciTech Premium Collection:**

(("self monitor" OR "self monitoring" OR "self monitors") OR ("self assess" OR "self assessed" OR "self assessment") OR ("self manage" OR "self managed" OR "self managing") OR ("self record" OR "self recorded" OR "self recording") OR “self surveillance” OR “patient* monitor*” OR ("measurement technologies" OR "measurement technology") OR “telemonitor*” OR ("remote monitoring") OR ("passive monitoring") OR ("active monitoring") OR “mood track*” OR “mood monitor*” OR ("experience sampling") OR “ecological momentary assessment”) NEAR/10 (("bipolar disorder" OR "bipolar disorders") OR ("manic depression" OR "manic depressive") OR “Bipolar affective psychos*” OR ("bipolar depression") OR “Manic disorder*” OR “depressi*” OR ("depressive disorder") OR “major depressive disorder*” OR “depression” OR ("affective disorder" OR "affective disorders") OR ("mood disorder" OR "mood disorders"))

http://nottingham.idm.oclc.org/login?url=https://www.proquest.com/search/2332894?accountid=8018

https://www.proquest.com/scitechpremium/

**Google incognito mode – first 200 results:**

No new papers identified

**Systematic Reviews reference checked:**

These are cited in the main paper

Supplementary Figure 2: Funnel plot for total adverse events prevalence meta-analysis
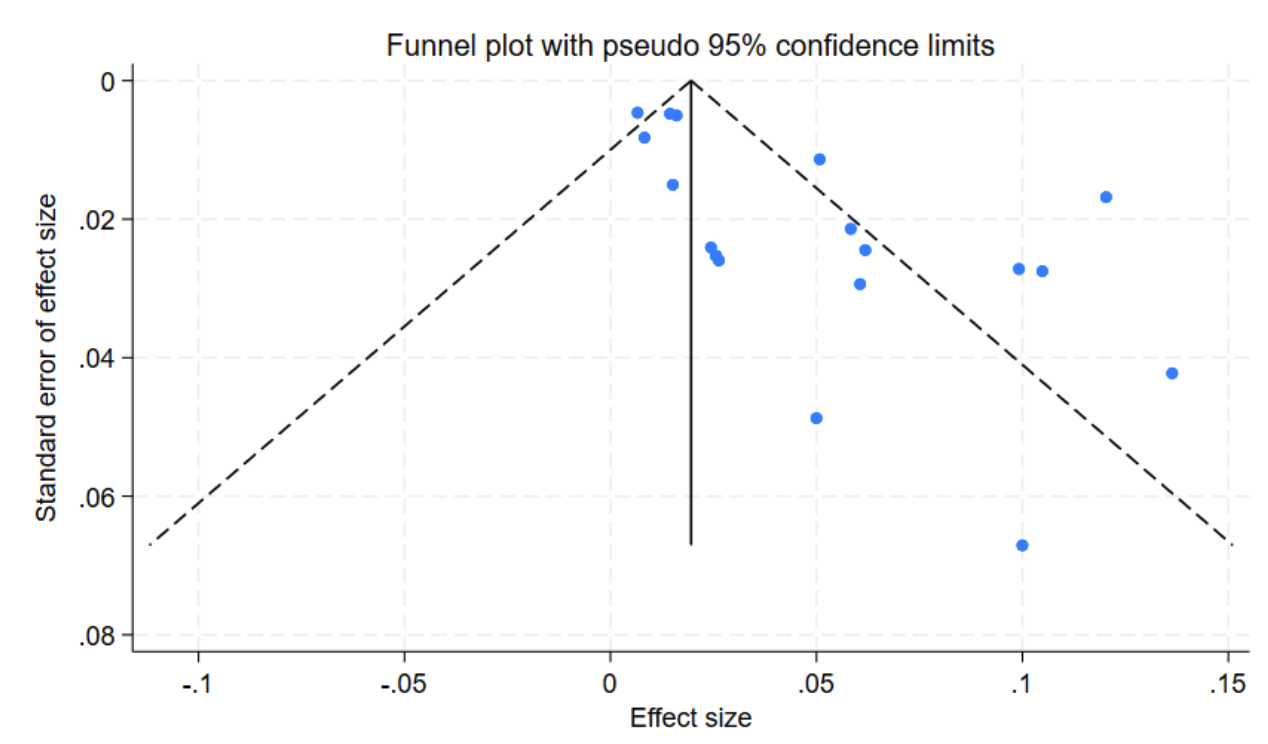


Supplementary Figure 3: Egger’s test for small study-effects


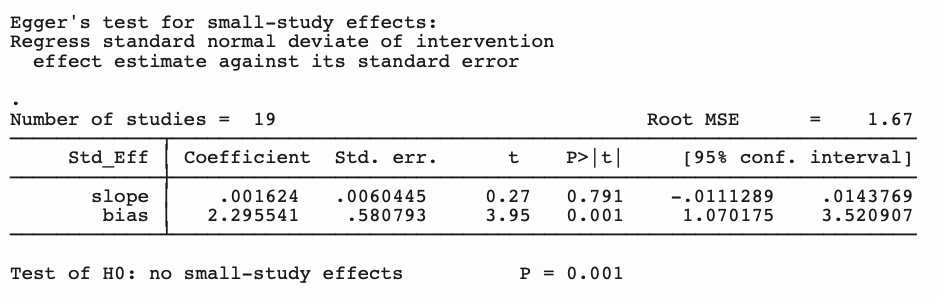

Supplement: Multimedia Appendix 1 [file mental-v12-e79500-s001.docx]
